# A microphysiological system HHT-on-a-chip platform recapitulates patient vascular lesions

**DOI:** 10.1038/s41467-026-76448-w

**Published:** 2026-08-13

**Authors:** Jennifer S. Fang, Christopher J. Hatch, Jillian Andrejecsk, William Van Trigt, Joshua R. Gonzales, Damie J. Juat, Edward K. Looker, Sophia R. Harris, Yu-Hsi Chen, Satomi Matsumoto, Abraham P. Lee, Christopher C. W. Hughes

**Affiliations:** 1 Department of Molecular Biology and Biochemistry, Charlie Dunlop School of Biological Sciences, University of California-Irvine, Irvine, CA USA; 2 Department of Cell and Molecular Biology, School of Science & Engineering, Tulane University, New Orleans, LA USA; 3 Department of Physiology, School of Medicine, Tulane University, New Orleans, LA USA; 4 Louisiana Cancer Research Center, New Orleans, LA USA; 5 Department of Biomedical Engineering, Henry Samueli School of Engineering, University of California-Irvine, Irvine, CA USA

**Keywords:** Tissue engineering, Mechanisms of disease

## Abstract

Hereditary Hemorrhagic Telangiectasia (HHT) is a rare congenital disease in which fragile vascular malformations (VM) focally develop in multiple organs. There are few treatment options and no cure. HHT patients inherit loss-of-function mutations affecting Alk1-Eng signaling; however, why this manifests as VMs remains poorly understood. Here we have developed a fully human cell-based microphysiological system of perfused vasculature in which inducible shRNA controls endogenous Alk1 in primary endothelial cells (EC). Resulting VMs develop over several days, recapitulate patient VM appearance, and require only a subpopulation of Alk1-deficient EC to trigger lesions. Single-cell transcriptomics suggests microvessel pruning and regression contribute to VM, while loss of PDGFB implicates mural cell recruitment. Finally, pharmacological VEGF/VEGFR inhibition blocks lesion formation. In summary, we have developed an HHT-on-a-chip model that faithfully reproduces HHT patient lesions and that can be used to better understand HHT disease biology and identify potential new HHT drugs.

## Abstract (web summary)

Hereditary Hemorrhagic Telangiectasia (HHT) is a rare genetic disease involving development of vascular malformations. Here, the authors present the VMO-HHT – an organ-on-a-chip model of HHT that produces vascular malformations like those seen in human HHT patients.

## Introduction

Hereditary Hemorrhagic Telangiectasia (HHT) is a rare congenital disease that impacts 1 in 5000 people (or possibly higher^1^) and is characterized by the sporadic development of vascular malformations^2,3^. These disorganized lesions include small, dilated, and fragile tangles of vessels called telangiectasias^4^ that affect skin and mucosa, especially in the nose, where their rupture leads to frequent and uncontrolled epistaxis (nosebleeds) that greatly impacts patient quality-of-life^5,6^. Many HHT patients also develop larger arteriovenous malformations (AVM)—enlarged arteriolar-to-venous shunts^7^—within their gut, lung, liver, and brain that can compromise tissue perfusion leading to organ damage and eventual failure^5^. HHT is subdivided into disease subtypes based on patient gene mutation status. Greater than 90% of HHT patients inherit loss-of-function mutations affecting either *ENG* (HHT1), which encodes Endoglin, or *ACVRL1* (HHT2), which encodes Activin-like Kinase receptor 1 (Alk1)^5^. Eng and Alk1 are endothelium-expressed cell surface TGF-β superfamily co-receptors, with Alk1 being the signal transducing component, and mutation of either affects Alk1-driven signaling through downstream Smads. Specifically, Alk1 receptor activation phospho-activates the Smad1/5 complex, which then binds to Smad4. The activated Smad complex translocates into the nucleus to regulate target gene expression^8^. A rare variant of HHT—juvenile polyposis-HHT (JP-HHT)^9^—accounts for ~2% of HHT cases and has been mapped to the *SMAD4* gene, a key integrator in the Smad pathway^9–11^. Importantly, patients are heterozygous for Alk1, Eng, or Smad4 mutations, with lesions being thought to occur as a result of somatic mutation of the residual allele^12–14^. A recent study that used deep sequencing of isolated HHT patient lesions has confirmed this hypothesis, but with the surprising additional observation that many of the lesions in the same patient are of mixed composition, including both heterozygous and subpopulations of homozygously-mutated endothelial cells (EC) containing different second hits—suggesting multiple loss-of-heterozygosity (LoH) events^13^. Relatedly, mouse studies have also shown that homozygous deletion of Endoglin in only a small subpopulation of EC is sufficient to induce AVMs when combined with a focal pro-angiogenic stimulus^15^.

The relevant ligands for the Alk1/Eng receptor complex are BMP9 and BMP10^16^, although other TGF-β ligands are also capable of binding at much higher concentrations^16^. Of these, BMP9 is present in blood at concentrations well above its binding affinity for the Alk1 receptor, suggesting that there is tonic signaling through this pathway in established vasculature, possibly to maintain EC quiescence^17,18^.

Unfortunately, there is currently no cure for HHT and few treatment options, due in part to the challenges of modeling HHT vascular malformations in the preclinical setting. Most research on the pathogenesis of vascular malformations has been conducted in genetically-modified mouse models wherein Alk1, Eng, or Smad4 are homozygously ablated, either globally or only in vascular endothelium^19^. Although these models have provided important insights into HHT disease biology^10,11,15,20–22^, AVMs occur only sporadically and can be difficult to identify and monitor in real-time. Several key insights have also been provided by zebrafish models, including the finding that Alk1 is a flow-sensitive gene and that dysregulated flow-directed EC migration may contribute to vascular malformation in HHT^23–26^. In this study, we use a microphysiological system (MPS) platform to generate a human cell-based in vitro microphysiological model of vascular malformations in HHT that can complement and enhance research in animal models.

MPS—or Organ-on-a-chip (OoaC)—refers to the engineering of artificial microtissues in a three-dimensional in vitro microenvironment that captures the microphysiology of native, intact tissue^27,28^. MPS models enable reproducible and high-throughput engineering of complex and physiological microtissues in an in vitro setting^29^. They are thus powerful new tools for understanding healthy and diseased tissues that will help in translational research by supporting both compound library drug screens and personalized medicine applications^27,30^.

We previously developed a human vessel-on-a-chip (Vascularized Micro-Organ, VMO) platform that combines primary human endothelial and perivascular cell types cultured in a three-dimensional hydrogel and in the presence of interstitial flow^30–33^. Under these conditions, EC self-organize over the course of 4–5 days into a lumenized microvascular network that is perfused by a gravity-driven blood substitute. We have previously used the VMO platform to study vascularized micro-tumors^31,34–36^, as well as to model healthy tissue-vascular interactions^32,37^. In this study, we describe an adapted VMO platform that allows for the development of disorganized vessel lesions with the architectural hallmarks of HHT telangiectasias and AVMs. We call this novel HHT-on-a-chip microphysiological platform the HHT-VMO and show that it can be used to provide valuable insights into HHT disease biology and to evaluate drugs for their therapeutic potential in HHT.

## Results

### Alk1-deficient EC exhibit altered EC behavior and form hyperdense microvasculature in vitro

As noted above, HHT patients are germline heterozygous for loss-of-function mutations that affect either Alk1 (*ACVRL1*) or its co-receptor Eng (*ENG*)^5^. To generate EC lacking intact Alk1 signaling, mimicking the LoH events thought to occur in lesions, we initially pursued a transient silencing RNA (siRNA) approach to target endogenous *ACVRL1* (si-*ACVRL1*) transcript. This achieved a near-complete knockdown of Alk1 mRNA that persisted for at least 7 days in primary human umbilical vein EC (HUVEC), but this knockdown fully recovered to control levels by day 12 (Supplementary Fig. 1a). We seeded EC transfected with si-*ACVRL1* (or non-targeting si-Ctrl) into our previously described VMO platform^31,32,34,38^ at equal starting cell densities (Supplementary Fig. 1b, c) and observed that microvasculature that formed from si-*ACVRL1* EC were hyperdense relative to si-Ctrl (Supplementary Fig. 1d–i). We observed a similar phenotype with siRNA knockdown of *ENG* (Supplementary Fig. 2), suggesting that this hyperdense phenotype arises from loss of signaling through the Alk1/Eng HHT-associated signaling pathway.

**Fig. 1:**
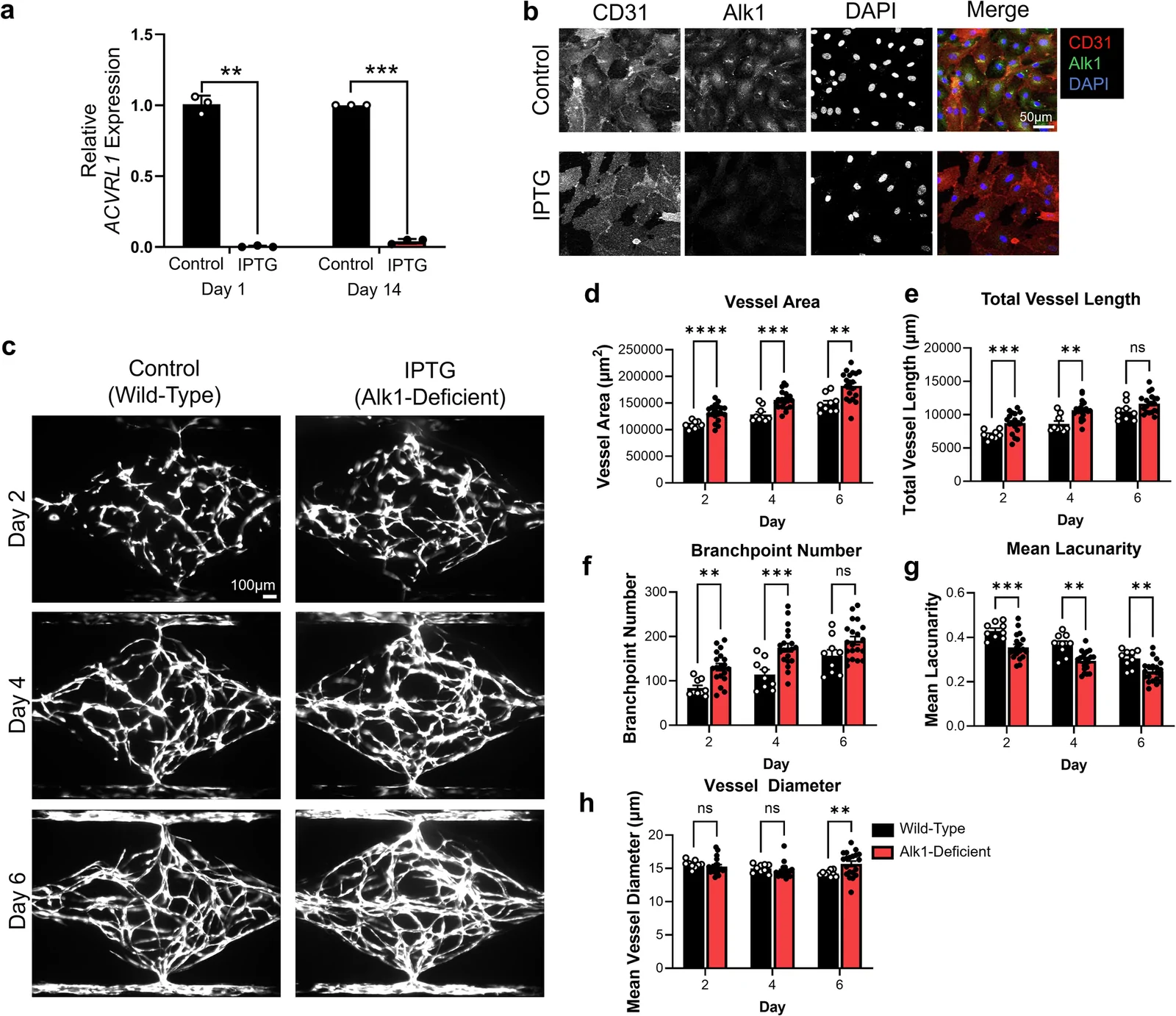
Alk1-deficient EC (via IPTG-sensitive shRNA) form hyperdense microvasculature in VMO. **a** IPTG eliminates >95% endogenous Alk1 mRNA expression in human EC at day 1 and day 14 post-transduction. (Sample size: *n *= 3 independent cultures for each group. Statistics: Two-tailed paired *t*-Test. *p*-values: Day 1: *p *= 0.001069, Day 14: *p *= 0.000108). **b** Endogenous Alk1 protein expression was also reduced in EC. **c** IPTG-treated Alk1-deficient EC form hyperdense microvasculature compared to untreated (i.e., no IPTG) Alk1-intact control EC. This was associated with **d** increased vessel area (*p*-values: Day 2: *p *< 0.0001, Day 4: *p *= 0.0009, Day 6: *p *= 0.0011), **e** increased total vessel length (*p*-values: Day 2: *p *= 0.0009, Day 4: *p *= 0.0037, Day 6: *p *= 0.1439), **f** increased branchpoint number (*p*-values: Day 2: *p *= 0.0092, Day 4: *p *= 0.0004, Day 6: *p *= 0.076, and **g** decreased mean lacunarity (*p*-values: Day 2: *p *= 0.0010, Day 4: *p *= 0.0057, Day 6: *p *= 0.0089). **h** Mean vessel diameter was increased, but only at the later timepoint. (Sample size: Wild-Type: *n *= 9, Alk1-Deficient: *n *= 20 independent devices. Statistics: Two-tailed 2-way ANOVA with mixed effects and adjustment for multiple comparison). All plotted data are presented as mean values ± standard error.

Next, to generate stable Alk1 knockdown that persists for longer than 7 days, we used lentiviral transduction to introduce an isopropyl ß-D-1-thiogalactopyranoside (IPTG)-sensitive *ACVRL1*-targeting short hairpin RNA (shRNA) into primary HUVEC at >85% transduction efficiency (Supplementary Fig. 3a, b). IPTG is an allolactose mimetic frequently used as an inducing agent to activate tunable transcription of genes under control of *lac* operon, such as in our IPTG-inducible sh-*ACVRL1* system. In resulting sh-*ACVRL1* EC, addition of IPTG for 48 h into the cell culture medium induced dose-dependent Alk1-deficiency (Supplementary Fig. 3c). 3 mM IPTG produced >90% *ACVRL1* mRNA knockdown, and transduction of the construct into EC was stable as evidenced by a persistent high-efficiency of *ACVRL1* gene knockdown at day 14 in response to IPTG (Fig. 1a). Importantly, we tested two independent shRNA sequences (Table 1) and results were indistinguishable (data not shown). Consequently, we used these constructs interchangeably in the reported studies. Alk1 protein expression was also reduced in sh-*ACVRL1* EC in response to IPTG (Fig. 1b).

**Table 1:** sh-*ACVRL1* clones and qPCR primers

sh-*ACVRL1* clones	
Name/gene name	Sequence
sh-*ACVRL1* Clone #1 (Sigma TRCN0000000355)	5′ – TCCAGAGAAGCCTAAAGTGAT – 3′
sh-*ACVRL1* Clone #2 (Sigma TRCN000000356)	5′ – CAGTCCAGAGAAGCCTAAAGT – 3′

qPCR primers	
*18S*	Forward 5′ – CCCCGGCCGTCCCTCTTA – 3′ Reverse 5′ – CGCCCCTCGATGCTCTTAG – 3′
*ACVRL1*	Forward 5′ – GCAACCTGCAGTGTTGCATC – 3′ Reverse 5′ – CGGATCTGCTCGTCCAGCAC – 3′
*EFNB2*	Forward 5′ – TATGCAGAACTGCGATTTCCAA – 3′ Reverse 5′ – TGGGTATAGTACCAGTCCTTGTC – 3′
*ENG*	Forward 5′ – TGCACTTGGCCTACAATTCCA – 3′ Reverse 5′ – AGCTGCCCACTCAAGGATCT – 3′

Next, we seeded sh-*ACVRL1* EC into our vessel-on-a-chip platform. Consistent with our initial experiments using siRNA (i.e. short-term Alk1 or Eng knockdown, Supplementary Figs. 1, 2), IPTG-treated (i.e. Alk1-deficient) sh-*ACVRL1* EC formed hyperdense microvasculature in our published VMO^31,35^ platform compared to untreated controls (Fig. 1c). This observed increase in microvessel density was quantified in FIJI/ImageJ as a significantly increased vessel area (Fig. 1d), increased total vessel length (Fig. 1e), increased branchpoint number (Fig. 1f), and a decreased mean lacunarity (Fig. 1g). At later times, overall vessel length was similar in control and knockdown, likely due to the physical constraints of the system. Perhaps related to this, vessel diameter was increased at later times (Fig. 1h). Importantly, IPTG alone had no effect on microvascular network formation from wild-type EC in the VMO or VMO-HHT (Supplementary Fig. 4). Interestingly, even though knockdown of Alk1 produced higher microvessel density and more enlarged vessels in the VMO, vessel network architecture remained relatively ordered in appearance, with no obvious development of disorganized focal lesions that resembled AVMs.

### HHT-VMO platform supports physiological flow at arteriolar levels

In mouse models of HHT, vascular malformations appear in the neonatal retina in regions associated with higher fluid shear stress, whereas at lower levels of shear, vascular overgrowth (telangiectasia-like) is seen^39^, suggesting that hemodynamic flow is a critical initiating signal for the development of these lesions. The critical contribution of hemodynamic flow to lesion formation in HHT is also supported by studies in zebrafish^23,26,40^. Using in silico approaches, we modeled flow in our VMO platform and found that a maximal fluid velocity of 2 μm/s, producing a maximal shear stress of <0.5 dynes/cm^2^ (Supplementary Fig. 5). These values are significantly lower than the flow velocity and shear stress values calculated for the regions predisposed to shunt formation in the retinal vasculature of neonatal mice^41^.

**Fig. 2:**
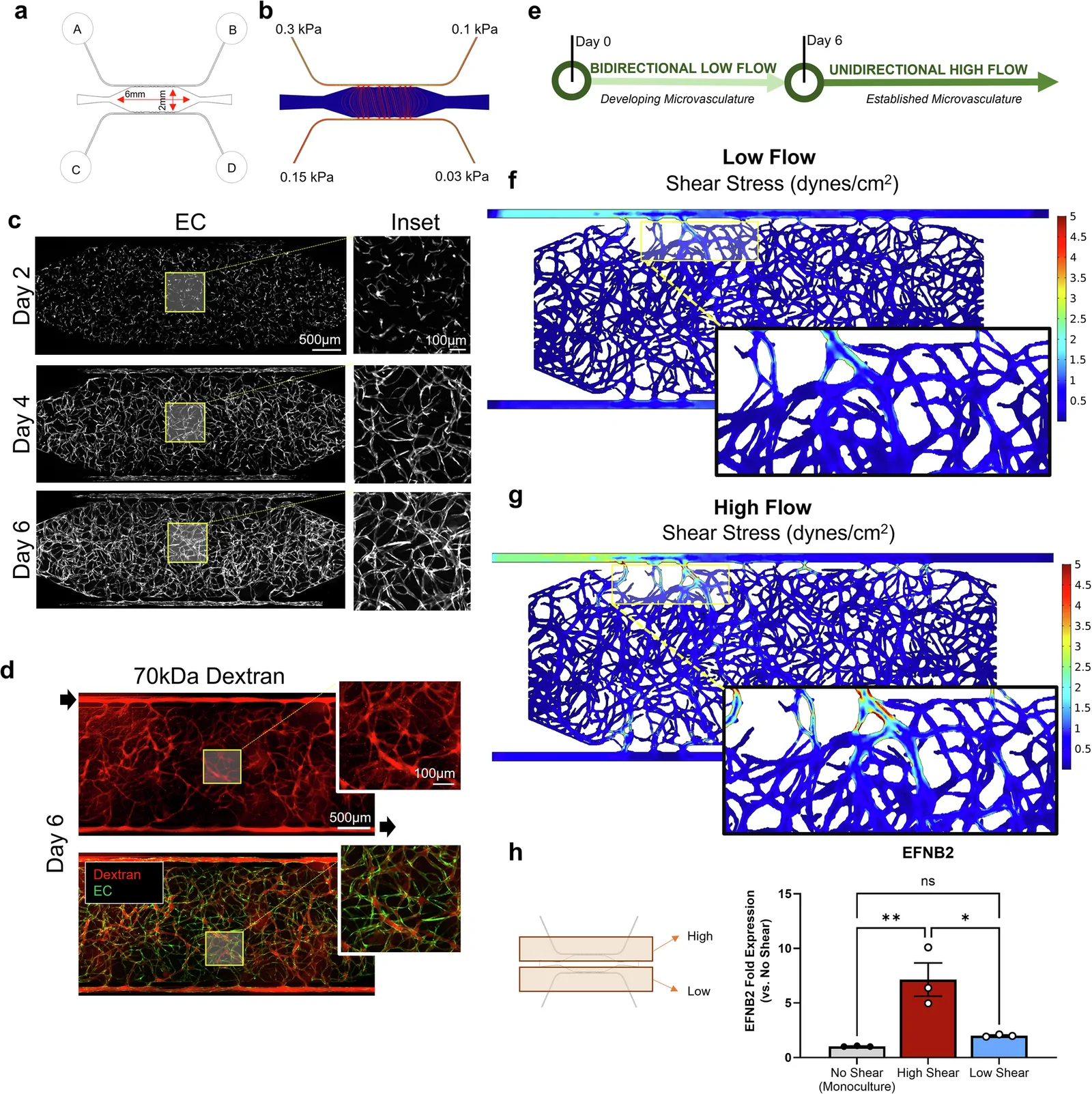
Modified microfluidic vessel-on-a-chip platform (HHT-VMO) supports formation of a perfused microvascular network under physiological shear. **a** Redesign of VMO platform to enlarge central tissue chambers and media reservoirs and to uncouple microfluidic channels. Flow is from A to B along the “artery” and C to D along the “vein”. Higher pressure on the arterial side drives net flow from A to D through the vasculature. **b** COMSOL modeling shows pressure differentials and interstitial flow lines prior to vessel establishment. **c** Seeding of fluorescent reporter-expressing primary human EC in the HHT-VMO under interstitial flow leads to the formation of perfused microvascular network over ~6 days (representative of *n *> 10 independent experiments). **d** Perfused microvessels are visualized by addition of a 70 kDa fluorescent dextran (red) to the media reservoirs. EC are green. (representative of *n *> 10 independent experiments). **e** Two-step flow protocol for inducing an established microvasculature. **f**, **g** In silico modeling of low and high flow settings produces regions of more physiological fluid shear stress (~5 dynes/cm^2^), particularly around the communication pores in the upper (arterial) media channel (insets). **h** Following 2 days exposure to high flow, EC in the high and low shear regions were isolated for qPCR analysis. EC in the high shear zone acquire expression of the arterial marker ephrin B2 (*EFNB2*). (Sample size: 3 independent cultures or devices per group. Statistics: Ordinary one-way ANOVA with adjustment for multiple comparisons. *p*-values: No Shear vs. High Shear: *p *= 0.0065, No Shear vs. Low Shear: *p *= 0.7264, High Shear vs. Low Shear: *p *= 0.0147). All plotted data are presented as mean values ±standard error.

To generate a platform with a more physiological range of intravascular flow and fluid shear stress, we adapted our base VMO^31,32^ by uncoupling the upper and lower microfluidic channels and enlarging the central tissue chamber and media reservoirs (Fig. 2a). This indeed led to higher levels of interstitial flow (Fig. 2b), but still in the range necessary to induce formation of a perfused microvascular network. Network formation took 6–8 days following initial cell seeding (Fig. 2c), consistent with how the microvasculature develops in our published VMO models^31,32,34,38^. Once vessel formation was complete, addition of 70 kDa fluorescent dextran to the media reservoirs marked lumenized microvessels and revealed that vessels are perfused and that flow is *only* through vessels (Fig. 2d). To model the normal developmental process, we next developed a two-step flow protocol to support the development of a perfused microvasculature under physiologic flow, wherein EC are initially exposed to bidirectional low flow for the first 6 days, mirroring the hemodynamic conditions of developing microvessels. After 6–8 days, networks were switched to unidirectional high (physiological) flow, consistent with the conditions present upon establishment of a systemic circulation in vivo (Fig. 2e). In silico modeling of low- (Fig. 2f) and high-flow (Fig. 2g) conditions confirms that they support physiologic fluid shear stress levels of up to ~1 dynes/cm^2^ and ~5 dynes/cm^2^, respectively. Importantly, we observed increased expression of the endothelial-specific arterial marker Ephrin B2 (*EFNB2*) only in regions of high shear stress following 2 days of high flow in the HHT-VMO platform (Fig. 2h). In parallel, perivascular cells in the high shear stress region of the microvasculature also acquire expression of smooth muscle α-Actin (SMA) (Supplementary Fig. 6), another indicator of vessel arterialization under these conditions.

### Alk1-deficient EC in the modified platform generate disorganized microvascular networks with lesions reminiscent of HHT patient telangiectasias

**Fig. 3:**
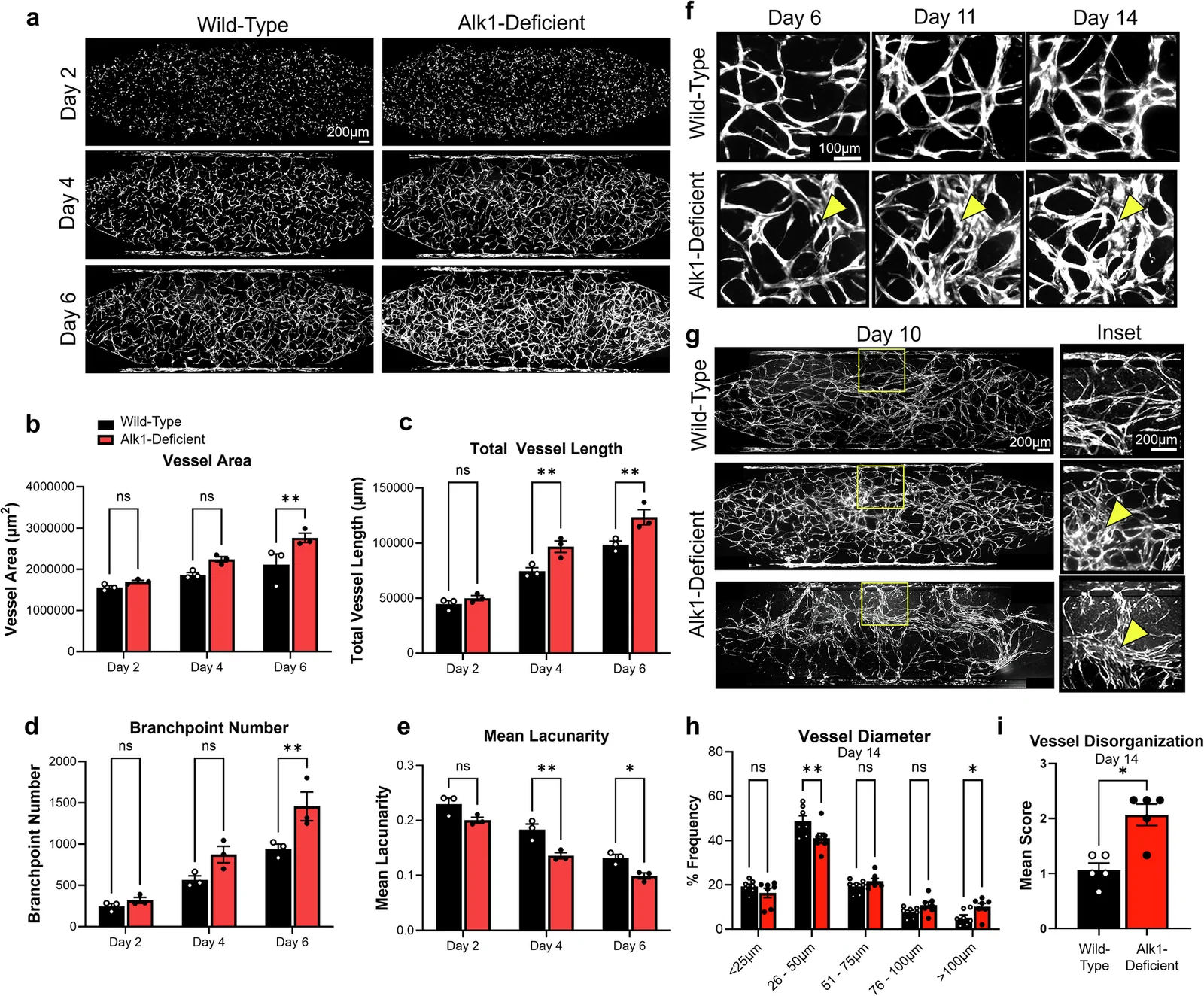
Alk1-deficient EC form hyperdense and disorganized microvasculature in the HHT-VMO platform. **a** Compared to control networks, IPTG-treated networks appear hyperdense during initial network formation, quantified by vessel morphometry as significant increases in **b** vessel area (*p*-values: Day 2: *p *= 0.8338, Day 4: *p *= 0.1475, Day 6: *p *= 0.0082), **c** total vessel length (*p*-values: Day 2: *p *= 0.7759, Day 4: *p *= 0.0096, Day 6: *p *= 0.0044), and **d** branchpoint number (*p*-values: Day 2: *p *= 0.9256, Day 4: *p *= 0.0921, Day 6: *p *= 0.0049). **e** Mean lacunarity was decreased by day 6 (*p*-values: Day 2: 0.0556, Day 4: *p *= 0.0026, Day 6: *p *= 0.0295). (Sample size: 3 independent devices per group. Statistics: Two-tailed 2-way ANOVA with adjustment for multiple comparisons). **f** IPTG-treated networks contained structures that continued to dilate with additional time in culture (yellow arrowhead). **g** By day 10, we observed networks with focally dilated lesions (middle), or a complete collapse of the microvascular network to a few dominant vessels (bottom). **h** At day 14, IPTG-induced Alk1-knockdown increased the frequency of vessels with a larger diameter (Sample size: 7 independent devices per group. Statistics: Two-tailed 2-way ANOVA with adjustment for multiple comparisons. *p*-values: <25 µm: *p *= 0.2141, 26–50 µm: *p *= 0.0015, 51–75 µm: *p *= 0.3038, 76–100 µm: *p *= 0.1659, >100 µm: 0.0356). **i** Semi-quantitative “vascular disorganization score” was increased in the absence of Alk1 (Sample size: *n *= 5 independent devices per group. Statistics: Two-tailed unpaired Mann–Whitney *t*-test. *p*-values: Wild-Type vs. Alk1-Deficient: *p *= 0.0238). All plotted data are presented as mean values ±standard error.

We generated EC transduced with the IPTG-sensitive sh-*ACVRL1* construct, which were then seeded into the HHT-VMO platform. IPTG was added to the medium beginning at day 0 and maintained throughout the timecourse of the experiment to induce knockdown of endogenous Alk1 expression. Wild-type (control) networks were not exposed to IPTG, thereby leaving Alk1 expression intact. Consistent with our observations in the original (low-flow) VMO platform (Fig. 1), IPTG-treated (Alk1-deficient) EC in the modified platform also formed hyperdense networks compared to wild-type controls when the shear was at the lower end of physiologic levels (Fig. 3a). This was quantified as a significantly increased vessel area, total vessel length and branchpoint number, as well as significantly decreased mean lacunarity, by day 6 (Fig. 3b–e). When networks were subsequently switched to high flow in the presence of IPTG, we observed microvascular structures that progressively grew over time (Fig. 3f), resulting in enlarged and dilated focal lesions that varied in their presentation from dense clusters or tangles to enlarged loop-like structures (Fig. 3g). This disorganized architecture is reminiscent of HHT patient telangiectasias, which are also highly variable and typically present in the nasal septum as “spots”, “loops”, “spiders”, or “raspberry”-like tangles of disorganized microvasculature^4^.

Consistent with the observed appearance of dilated telangiectasia-like lesions with addition of circulating IPTG, morphometric analysis of the entire microvascular network revealed a significant increase in the frequency of vessels with a diameter >100 µm and a concomitant decrease in the frequency of vessels with a diameter <50 µm (Fig. 3h). Using an investigator-blinded semi-quantitative scoring approach (see section “Materials and methods”), we also detected a significant increase in vessel disorganization score in IPTG-treated networks (Fig. 3i). Importantly, there was no effect on vessel network appearance when wild-type (i.e., untransduced) EC (Supplementary Fig. 4e–h) were treated with IPTG in the platform.

### Lesion formation only occurs when Alk1 is lost early in vessel development

**Fig. 4:**
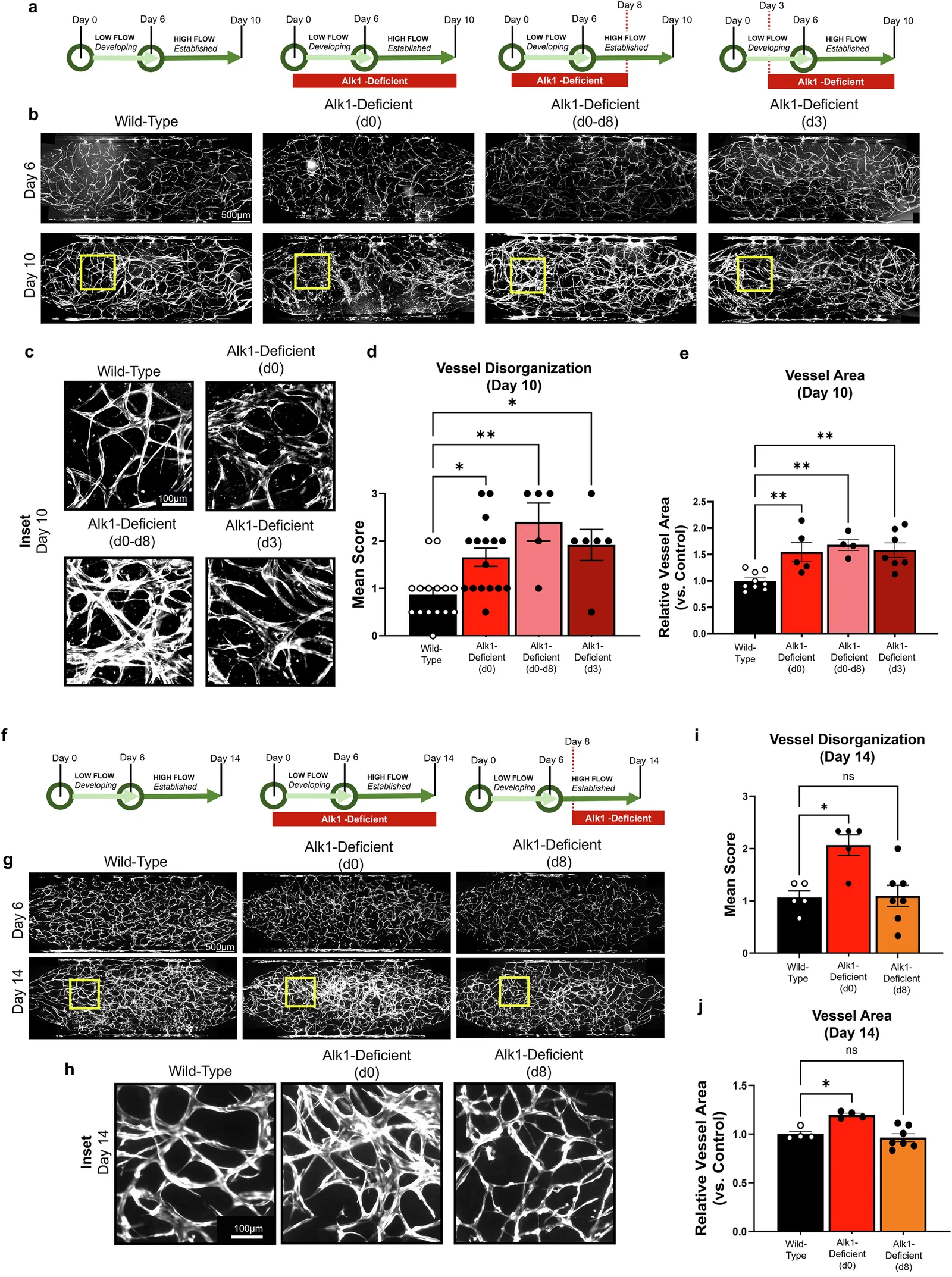
Alk1 knockdown in HHT-VMO during (but not after) network formation results in microvascular lesions. **a** IPTG was administered to initiate Alk1 knockdown at days 0 or 3, during microvascular network formation, and maintained throughout the length of the experiment or removed at day 8 (i.e., d0–d8) once microvascular networks were fully established. **b** In all conditions, Alk1 knockdown during the early “developing” phase of microvascular network establishment resulted in the appearance of disorganized microvascular lesions. **c** All insets reflect the same region in the upper middle of their respective networks as denoted by the yellow box in (**b**). **d** Vessel disorganization scores (Sample Size: Wild-Type: *n *= 15, Alk1-Deficient (d0): *n *= 16, Alk1-Deficient (d0–d8): *n *= 6, Alk1-Deficient (d3): *n *= 5 independent devices, Statistics: Kruskal–Wallis Test with adjustment for multiple comparisons. *p*-values: Control vs. d0: *p *= 0.0199, Control vs. d0–d8: *p *= 0.0030, Control vs. d3: *p *= 0.0334) and **e** relative total vessel area measurements were significantly elevated at Day 10 in all treatment groups compared to control (Alk1-intact) network (Sample Size: Wild-Type: *n *= 9, Alk1-Deficient (d0): *n *= 5, Alk1-Deficient (d0–d8): *n *= 4, Alk1-Deficient (d3): *n *= 7 independent devices, Statistics: Ordinary one-way ANOVA with adjustment for multiple comparisons. *p*-values: Control vs. d0: *p *= 0.0094, Control vs. d0–d8: *p *= 0.0028, Control vs. d3: *p *= 0.0023). Next, **f** IPTG was administered to knockdown Alk1 only after networks were fully established and perfused at day 8. **g** IPTG exposure beginning at day 8 failed to produce lesions by day 14. **h** All insets reflect the same region in the upper middle of their respective networks as denoted by the yellow box in (**g**). **i** Vessel disorganization score (Sample Size: Wild-Type: *n *= 5, Alk1-Deficient (d0): *n *= 5, Alk1-Deficient (d8): *n *= 7 independent devices, Statistics: Kruskal–Wallis Test with adjustment for multiple comparisons. *p*-values: Control vs. d0: *p *= 0.0299, Control vs. d8: *p *> 0.9999) and **j** total vessel area was not significantly changed at day 14 in IPTG d8 networks compared to control (Alk1-intact) networks (Sample Size: Wild-Type: *n *= 4, Alk1-Deficient (d0): *n *= 4, Alk1-Deficient (d8): *n *= 7 independent devices, Statistics: Ordinary one-way ANOVA with adjustment for multiple comparisons. *p*-values: Control vs. d0: *p *= 0.0106, Control vs. d8: *p *= 0.7124). (Note: the data in Fig. 3i are a subset of these data). All plotted data are presented as mean values ±standard error.

To assess when Alk1 expression is most critical during vessel development, we seeded sh-*ACVRL1* EC into the HHT-VMO and applied IPTG to the perfusion medium prior to network formation (day 0, as described above), during active network formation (beginning at day 3, or beginning at day 0 but lasting only until day 8), or after the vasculature was largely established (day 8). We then assessed network morphology at days 10 and 14, to allow a minimum of 6 days in each group for lesions to develop after onset of IPTG; these timepoints were selected based on our earlier studies (Fig. 3) showing that lesions became obvious by day 6 when IPTG was administered at day 0. Remarkably, only the administration of IPTG at day 0 or day 3 led to the appearance of disorganized vascular lesions, even if IPTG was subsequently removed after day 8 (Fig. 4a–e). By contrast, addition of IPTG beginning at day 8 did not produce lesions (Fig. 4f–j). This indicates that there is a critical early window during vessel network development when the absence of Alk1 can trigger lesion formation. In the HHT-VMO system, this window is prior to 8 days. These findings are consistent with work from the Oh lab^15,20,21^, which has used mouse models of HHT to show that loss of Alk1 expression drives lesion formation in the early phases of an angiogenic response, whereas loss of Alk1 in stable vessels in the absence (or neutralization) of pro-angiogenic signaling does not produce a phenotype.

### Enrichment for strongly Alk1-deficient EC drives lesions reminiscent of arteriovenous malformations

**Fig. 5:**
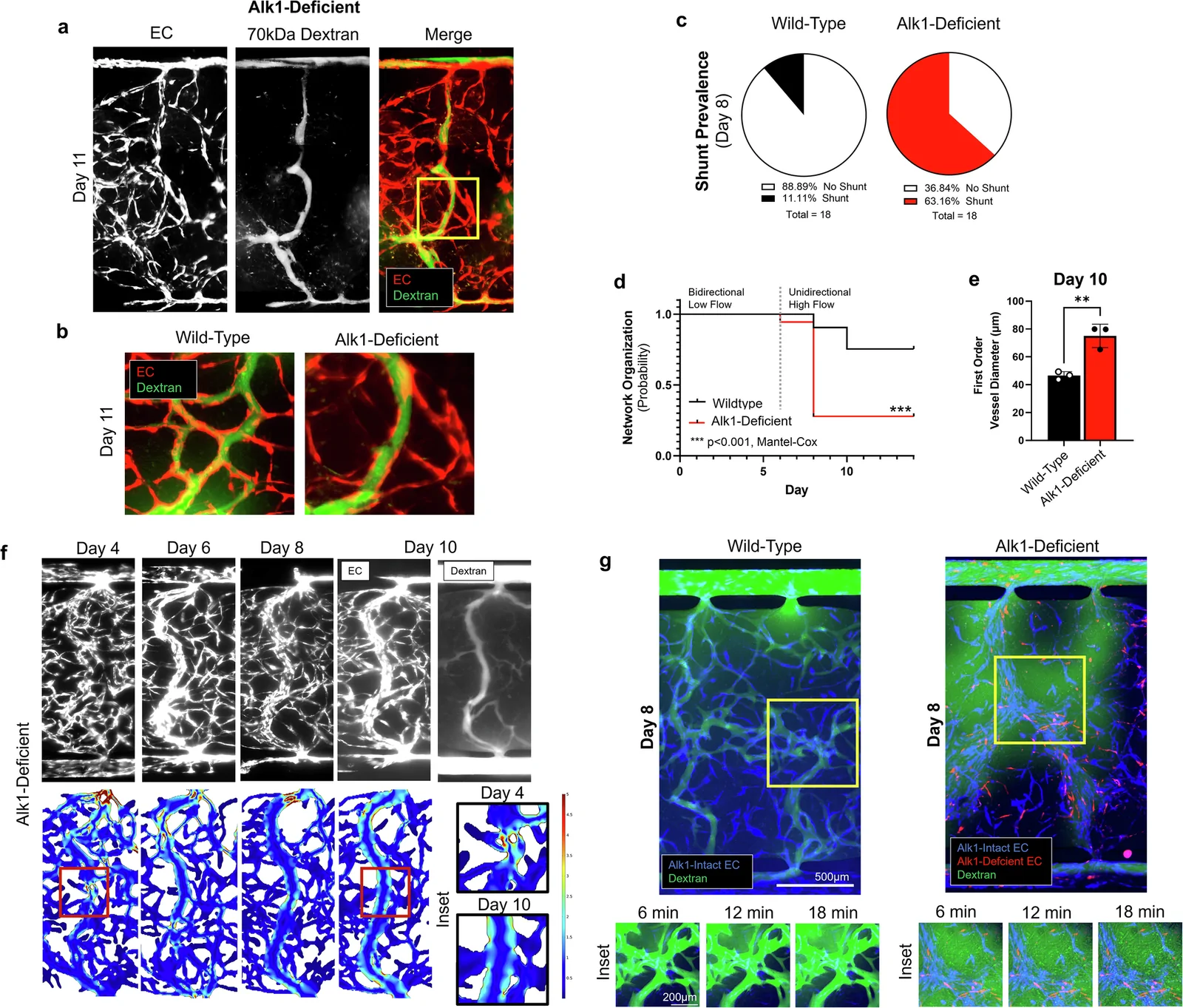
Enrichment for Alk1-deficient EC drives formation of shunt-like structures. **a** sh-*ACVRL1* EC were purified using puromycin pre-selection and then co-seeded with untransduced (Alk1-intact) EC. Under these conditions, Alk1-deficient networks developed enlarged shunt-like structures by day 11 that directly connected upper and lower microfluidic channels. **b** Intravascular flow (marked by perfusion of 70 kDa fluorescent dextran (green)) bypassed adjacent microvessels. **c** Shunt-like structures were observed in 63.2% of Alk1-deficient networks, compared to only 11.1% of wild-type networks. **d** Shunt-like structures were evident by day 8, after switching from bidirectional low-flow to unidirectional high-flow conditions. (Sample size: Wild-type: *n *= 21, Alk1-deficient: *n *= 18 independent devices, Statistics: Mantel–Cox log-rank test, *p *= 0.0003). **e** Mean vessel diameter of shunt-like structures was significantly increased compared to first-order vessels in the same region of wild-type networks. (Sample size: *n *= 3 independent devices per group, Statistics: Unpaired Students’ *t* Test, *p *= 0.0052). **f** In silico modeling of intravascular flow revealed that dilated lesions were associated with high shear stress in downstream branching vessels, which subsequently enlarged and appeared to anastomose over time to form the complete shunt. **g** Perfused 70 kDa dextran rapidly entered the extravascular space in Alk1-deficient networks compared to wild-type controls, where dyes persisted within vessel lumens with minimal extravascular leakage for up to 18 min, indicating that shunts have greatly reduced barrier function compared to healthy (Alk1-intact) vessels. All plotted data are presented as mean values ± standard error.

The approach described above reproducibly produced disorganized and dilated lesions reminiscent of HHT patient telangiectasias, but shunt-like structures did not reliably develop. We hypothesized that this might be because Alk1-deficiency is heterogeneous across the transduced EC population due to the relatively low MOI used (see section “Materials and methods”). Therefore, to enhance consistency, we added a puromycin enrichment step prior to cell seeding in the HHT-VMO (or in a modified version of the platform redesigned to fit a standard 96-well plate format while keeping all other parameters consistent, Supplementary Fig. 7). To mimic the in vivo situation, where only some EC are homozygous mutant due to an LoH event^13^, we co-seeded wild-type EC along with purified sh-*ACVRL1* EC in a one-to-one ratio. Remarkably, we now observed that on addition of IPTG and exposure to our two-step flow protocol (Fig. 2e), we could consistently develop shunt-like structures following exposure to high flow conditions (Fig. 5). Conduits were especially obvious when intravascular flow was marked by perfusion of 70 kDa fluorescent dextran, which flowed directly from the upper (arteriole-like) to the lower (venule-like) microfluidic channels, through these enlarged vascular conduits (Fig. 5a).

A key feature of a developing AVM is that due to its increasing diameter, it carries a greater fraction of the available blood and thereby starves adjacent branching microvessels. These branching collateral microvessels eventually regress, leaving an enlarged direct artery-to-vein connection—an AVM—in place of the previous balanced capillary bed. We observed a similar phenomenon in the HHT-VMO, and again, this was particularly apparent when vessels were perfused with fluorescently labeled dextran. Shunt-like vessels were strongly fluorescent, whereas the adjacent capillary-like vessels became extremely narrow and appeared to cease supporting flow (Fig. 5b). Disorganized, shunt-like lesions formed in 63.2% of IPTG-treated networks, compared to only 11.1% of control networks (Fig. 5c), and these vascular structures typically formed by day 8, two days after networks were switched from bidirectional low flow to unidirectional high flow (Fig. 5d). In the retina of Alk1 mutant mice, shunts develop preferentially in first-order retinal vessels—i.e., vessels that branch immediately from the primary arteries that feed retina—that are exposed to high fluid shear^39^. Consistent with this finding, we also observed that shunts in the HHT-VMO appeared more reliably in first-order vessels in HHT-VMO networks—i.e., the first branching vessels immediately downstream from the upper microfluidic channel where fluid velocity and shear stress is calculated to be highest (Fig. 2f, g). These shunt-prone first-order vessels were measured at day 10 for their vessel diameter at the upper, middle, and lower regions of the tissue chamber. In networks containing Alk1-deficient EC, the mean diameter of shunt-like structures was 75.0 ± 19.1 μm, compared to 46.6 ± 11.7 µm in Alk1-intact (i.e., no IPTG) control networks (Fig. 5e). This corresponds to a 61% increase in first-order vessel diameter in Alk1-deficient vessel networks compared to wild-type controls, and a 20-fold increase in flow, consistent with the development of a shunt.

To better understand the progression by which first-order vessels enlarge into shunt-like conduits in Alk1-deficient networks, we imaged fluorescent vessels to track shunt formation over time. At day 4, overall microvascular architecture appears relatively organized, although focal, disorganized telangiectasia-like structures—if not shunts—are already apparent. This is consistent with the early hyperdense phenotype we reported at this timepoint in Fig. 3. Interestingly, when we used in silico modeling to understand how intravascular flow is affected by the presence of these telangiectasia-like lesions, we found that flow (and thus fluid shear stress) was variable throughout the length of these first-order vessels (Fig. 5f, inset) Specifically, we found that shear is elevated immediately downstream of a dilated, telangiectasia-like tangle (Fig. 5f, day 4 inset). By day 10, we found that these high-shear regions had outwardly remodeled to increase vessel diameter resulting in reduced and more homogenous shear—a normal vascular response to high focal flow and shear to redistribute flow primarily through these large shunt-like conduits—resulting in an apparent AVM (Fig. 5f, day 10 inset). Further studies are needed to confirm this finding at greater time- and fluorescent-imaging resolution, as well as altered expression of arteriovenous markers.

Lastly, we tested if the shunt-like structures that formed in the VMO-HHT are more permeable to circulating fluid. At Day 8, we circulated 70 kDa fluorescent dextran through shunt-like lesions that formed in (IPTG-treated) Alk1-deficient networks and monitored dextran leak into the intravascular space over 18 min. Whereas fluorescent dextran was largely retained within the vessel lumens of wild-type networks, we observed that dextran leaked rapidly across the vessel wall of shunt-like structures, suggesting reduction in vascular barrier function in these lesions (Fig. 5g).

### Shunts are comprised of both Alk1-intact and Alk1-deficient EC

A reasonable hypothesis regarding lesion formation in HHT is that a single clone of HHT-mutant EC expands to dominate a region of the vascular network, and that this region undergoes AVM formation. This has been previously observed to drive vascular malformation in diseases such as Cerebral Cavernous Malformation (or CCM)^42^. An alternative hypothesis, however, supported by sequencing studies of HHT lesions from human patients is that lesions are heterogeneous for the presence of bi-allelically mutant cells, and this appears to be true for all three major HHT subtypes.^13,43–45^. This is consistent with an alternative, non-clonal mechanism of lesion formation.

**Fig. 6:**
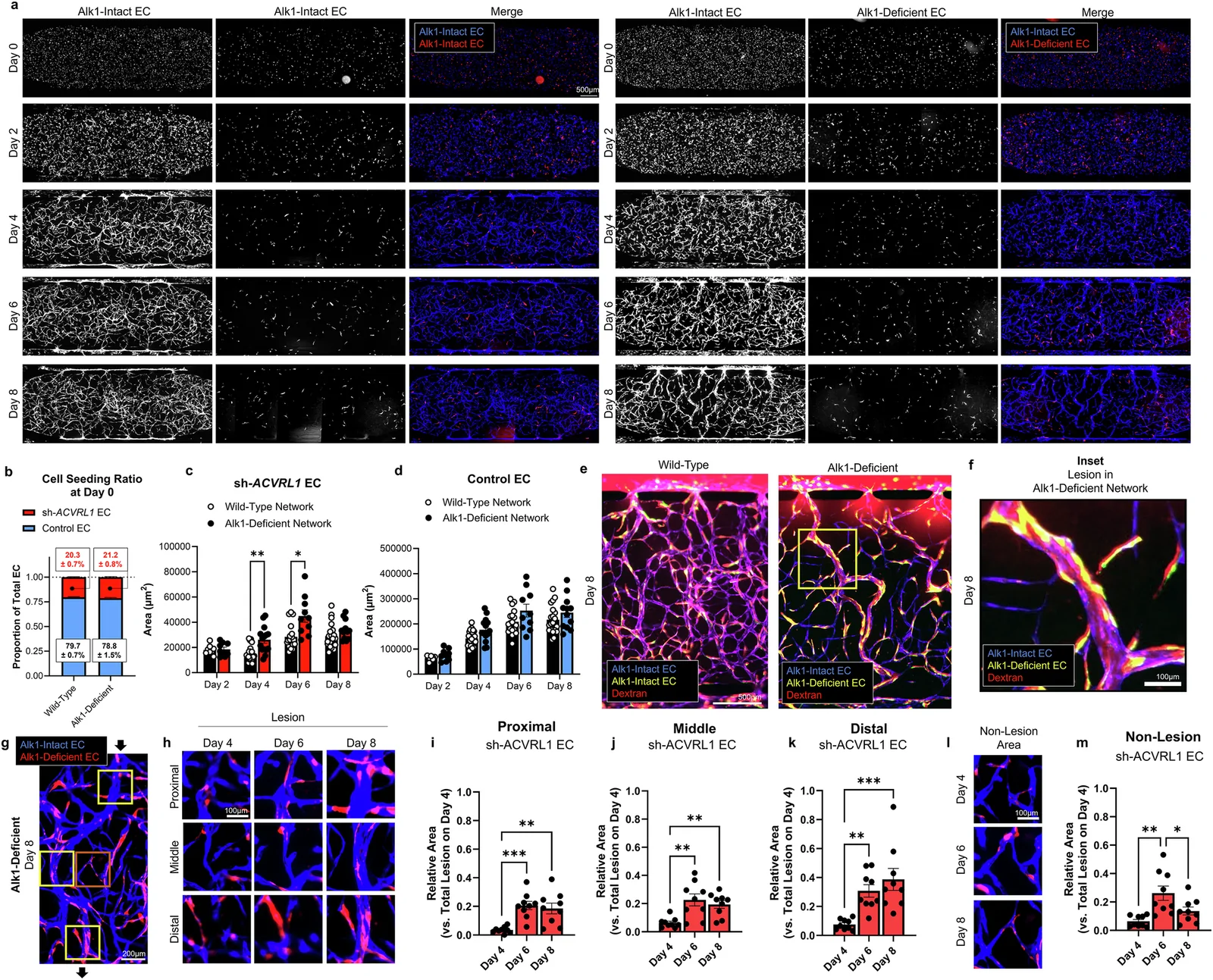
Lesions are chimeric structures comprised of both Alk1-intact and Alk1-deficient EC. **a** Co-seeding of Alk1-intact (i.e., untransduced, blue) with Alk1-deficient (i.e., IPTG-sensitive sh-*ACVRL1* red) EC expressing distinct fluorophores enable tracking of each cell population as networks develop. **b** Control and sh-*ACVRL1* EC were co-seeded at a 5:1 ratio, and networks were treated (or not) with IPTG to knockdown Alk1 in sh-*ACVRL1* cells only. (Sample size: Wild-Type: *n *= 3, Alk1-Deficient: *n *= 5 independent devices). **c** Alk1-deficient EC undergo an early expansion of the EC population at Days 4 and 6. (Sample size: Day 2: Wild-type network: *n *= 8, Alk1-Deficient network: *n *= 9, Day 4: Wild-type network: *n *= 23, Alk1-Deficient network: *n *= 15, Day 6: Wild-type network: *n *= 18, Alk1-Deficient network: *n *= 10, Day 8: Wild-type network: *n *= 20, Alk1-Deficient network: *n *= 12 independent devices, Statistics: Two-tailed 2-way ANOVA with mixed effects and adjustment for multiple comparisons. *p*-values: Day 2: *p *= 0.9995, Day 4: *p *= 0.0048, Day 6: *p *= 0.0364, Day 8: *p *= 0.4464). By contrast, **d** Alk1-intact (control) EC undergo a more gradual expansion irrespective of the presence of IPTG (Sample size: Day 2: Wild-type network: *n *= 8, Alk1-Deficient network: *n *= 9, Day 4: Wild-type network: *n *= 23, Alk1-deficient network: *n *= 14, Day 6: Wild-type network: *n *= 18, Alk1-Deficient network: *n *= 10, Day 8: Wild-type network: *n *= 20, Alk1-Deficient network: *n *= 12 independent devices, Statistics: Two-tailed 2-way ANOVA with mixed effects and adjustment for multiple comparisons. *p*-values: Day 2: *p *= 0.5235, Day 4: *p *= 0.0953, Day 6: *p *= 0.4475, Day 8: *p *= 0.6476). **e** Shunt-like lesions are comprised of both Alk1-intact and Alk1-deficient EC. IPTG was added to the “Alk-deficient” panel to induce Alk1 shRNA in the yellow cells. Blue cells contain a control shRNA. **f** Inset of (**e**). **g** Area analysis of Alk1-intact (blue) and Alk1-deficient (red) EC in proximal, middle, or distal regions of a developing shunt (yellow boxes). **h** Insets of yellow boxes in (**g**). **i**–**k** Lesions expand in part due to significantly increased contributions of Alk1-deficient EC, which appear especially abundant in distal (i.e., low flow) regions of the shunt (Sample size: *n *= 9 independent device lesions, Statistics: Ordinary one-way ANOVA with adjustment for multiple comparisons, *p*-values: panel (**i**): Day 4 vs. Day 6: *p *= 0.0004, Day 4 vs. Day 8: 0.0016, panel (**j**): Day 4 vs. Day 6: *p *= 0.0015, Day 4 vs. Day 8: *p *= 0.0098, panel (**k**): Day 4 vs. Day 6: *p *= 0.0066, Day 4 vs. Day 8: *p *= 0.0004). **l** Similar analysis of adjacent non-lesion vessels (inset from brown box in (**g**)) reveals (**m**)) Alk1-deficient EC undergo a transient expansion and loss in non-lesion areas (Sample size: *n *= 9 independent device non-lesion regions, Statistics: Ordinary one-way ANOVA with adjustment for multiple comparisons, *p*-values: Day 4 vs. Day 6: *p *= 0.0012, Day 4 vs. Day 8: *p *= 0.3080, Day 6 vs. Day 8: 0.0406). All plotted data are presented as mean values ± standard error.

To test in our platform whether HHT lesions arise from cell autonomous or cell non-autonomous mechanisms, we expressed distinct fluorescent reporters in wild-type (Alk1-intact) and sh-*ACVRL1* (Alk1-deficient) EC populations in the HHT-VMO, and co-mixed the cells in a 5:1 ratio (Fig. 6a). We applied IPTG (or not) beginning at day 0 to induce Alk1 knockdown selectively in sh-*ACVRL1* EC. Interestingly, we observed that shunt-like lesions robustly formed in IPTG-treated networks even when Alk1-deficient EC represented only 20% of all EC (Fig. 6b)—or even less than 8% (Supplementary Fig. 8) as has been previously reported for human lesions^13^. We tracked each cell population over time as lesions developed and observed that Alk1-deficient EC undergo an acute expansion at Days 4–6 (Fig. 6c) that was not observed for Alk1-intact EC (Fig. 6d). This increase in Alk1-deficient EC may be due to either previously reported effects of Alk1-deficiency to increase both EC cell size (Supplementary Fig. 9) and cell proliferation (Supplementary Fig. 10), which we also confirmed occurs with Alk1 knockdown in our cells. Alternatively, this may also be related to defects in flow-dependent EC migration, as has been previously reported in animal models^23,26,46^. Importantly, no aberrant vessel morphology was observed when EC transduced with control vector were co-mixed with wild-type EC (Supplementary Fig. 11).

To assess the EC composition of lesions and because only rhodamine- or FITC-conjugated dextrans were available, EC were imaged on red and green channels first, and then immediately re-imaged following administration of 70 kDa rhodamine dextran to perfusing media to mark intravascular flow. Pre- and post-dextran images were then stitched together, manually overlaid, and differentially false-colored in order to distinguish each (Alk1-intact vs. Alk1-deficient) EC population from the other and from the perfused fluorescent dextran. In control networks (lacking IPTG) wherein both EC populations (blue and yellow, Fig. 6e) express Alk1, EC were integrated evenly into the network at day 8 and neither telangiectasia-like, nor AVM-like vascular lesions developed. Instead, all microvessels were well perfused as determined by distribution of circulating 70 kDa fluorescent dextran (Fig. 6e). Consistent with the observed mosaicism of human HHT lesions^13^, addition of IPTG to knockdown Alk1 in 20% of EC (Alk1-deficient, yellow) resulted in chimeric shunts compromised of an intermingling of blue (Alk1-intact, wild-type) and yellow (Alk1-knockdown) EC (Fig. 6e, f, inset).

To further explore how Alk1-deficient EC integrate into HHT lesions as they develop, we examined the EC composition in proximal (i.e., high flow), middle, and distal (i.e., low flow) regions of the shunt over time from day 4 (prior to the appearance of lesions) to day 8 (after lesions are apparent) (Fig. 6g, h). In all regions, we quantified a significant expansion of vessel area due to both increases in Alk1-intact (blue) and Alk1-deficient (red) EC; however, Alk1-deficient (red) EC appeared to be especially prevalent in the distal (i.e., low flow) regions of the shunt compared to other areas (Fig. 6i–k). By contrast, we observed that in adjacent non-lesion vessels, Alk1-deficient EC undergo a transient expansion at Day 6 followed by a loss at Day 8 perhaps related to the regression of these nearby microvessels (Fig. 6l, m).

### Gene expression changes in Alk1-deficient microvasculature

**Fig. 7:**
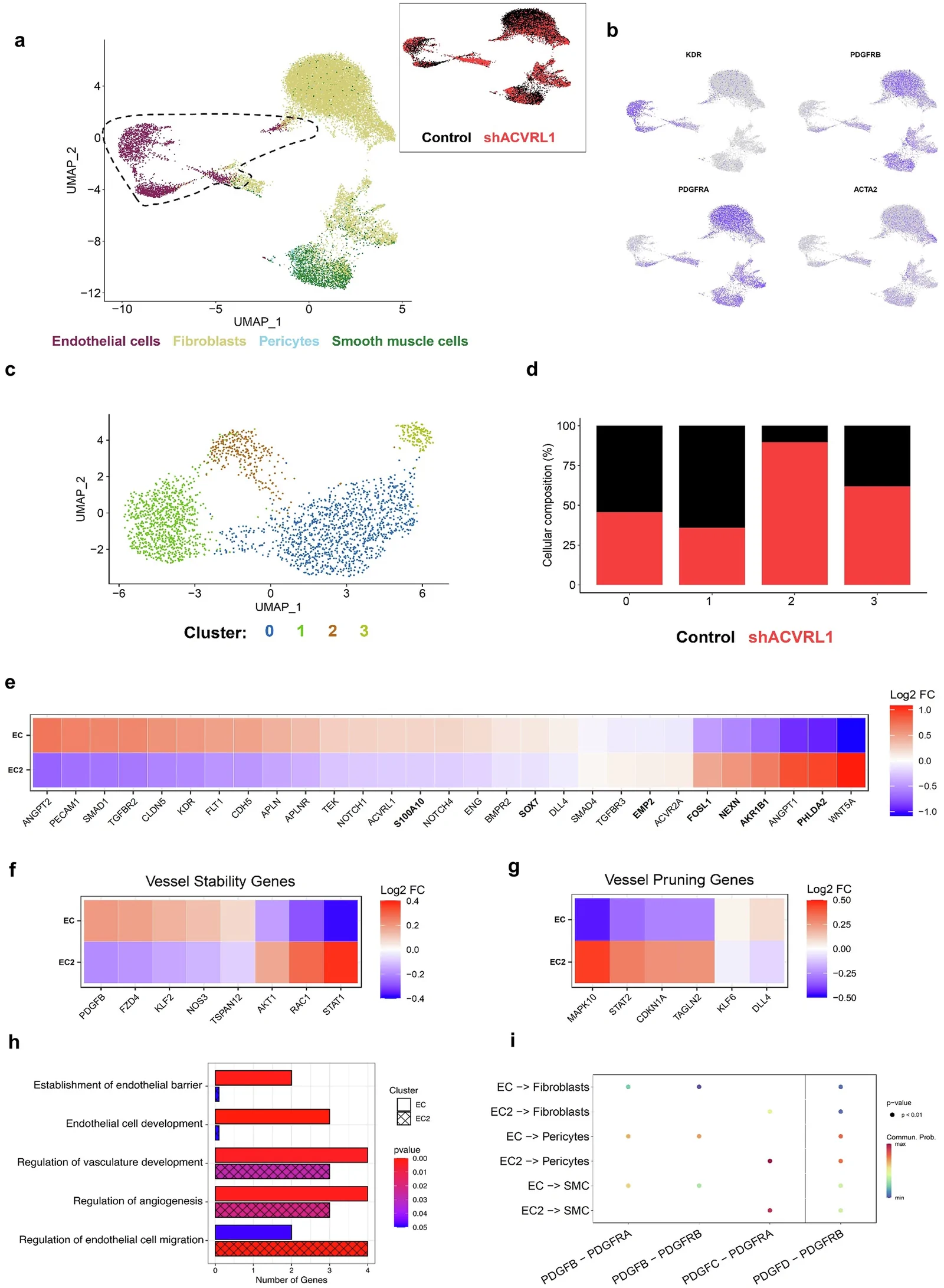
Identifying endothelial cell heterogeneity in control and shACVRL1 vascular micro-organs. Single-cell RNA sequencing of the control and shACVRL1 datasets shows **a** that each population contains endothelial cells, fibroblasts, pericytes, and smooth muscle cells (subset) with certain regions being unique to either dataset. **b** Expression of common marker genes distinguishes endothelial cells from the stromal population. **c** Subsetting of endothelial cells (dashed outline in (**a**)) and integration. Unsupervised clustering shows four distinct clusters. **d** Diseased is mainly comprised of endothelial cells from the shACVRL1 dataset. **e** Pathway analysis showing top unique genes for the Diseased-EC (i.e., ‘EC-2’) cluster. **f** Heatmap showing decreased expression of vessel stabilization genes in Diseased-EC^48^. **g** Heatmap showing increased expression of vessel pruning genes in Diseased-EC^48^. **h** Bar plot of pathway analysis on the top DEGs (presented in Supplementary Data 1, identified using DESeq2 method) for Diseased-EC and Control-EC, showing the number of genes in the DEGs for each pathway as well as the *p*-value for the pathway as determined by Fischer’s exact test using EnrichR. (DESeq2 tests for differential expression by use of negative binomial generalized linear models, where the estimates of dispersion and logarithmic fold changes incorporate data-driven prior distributions). **i** Cell-cell communication for EC to stroma evaluating PDGF signaling.

To better understand gene expression changes in Alk1-deficient EC that may contribute to lesion formation, we generated control networks and mosaic networks comprised of both Alk1-intact and Alk1-deficient EC (as described in Fig. 6) and performed single-cell RNA sequencing (scRNAseq) (Supplementary Fig. 12a–d) after lesions were confirmed to have formed on Day 8. We identified four distinct cell type clusters, including cells with gene expression signatures consistent with EC, pericytes, smooth muscle cells and fibroblasts (Fig. 7a, b). All four populations were observed in both control and IPTG-treated (Alk1-deficient) networks (Fig. 7a, subset). Sub-clustering of only EC (11% of all sequenced cells in control networks, 14% of all sequenced cells in IPTG-treated networks) revealed four distinct cell clusters (Fig. 7c, d) which we were able to identify as i) Shear-responsive, ii) Remodeling, 3) Diseased, and 4) Cycling EC based on significant changes in gene enrichment signaling pathways related to the above (Fig. 7e). In particular, the Diseased EC cluster had reduced expression of *ACVRL1* (Alk1)—as well as reduced expression of *ENG* (Endoglin)—(data not shown) and was enriched in IPTG-treated (Alk1-deficient) networks (Fig. 7f).

Diseased (*ACVRL1*/Alk1-deficient) EC exhibited significant changes in gene enrichment for signaling pathways related to tissue growth and artery morphogenesis (Fig. 7e). Closer analysis of differentially expressed genes (DEGs) in Diseased EC (vs. all other EC, Supplementary Data 1) revealed that several genes overexpressed in telangiectasias from HHT2 patients^47^ were also upregulated in Diseased EC (compared to all other EC, i.e., “Control” EC). These include EMP2, FOSL1, NEXN, AKR1B1, and PHLDA2 (Fig. 7g). We also saw down-regulation of several pro-angiogenic genes, including ANGPT2, KDR, FLT1 and DLL4, which at first seemed surprising given the vascular overgrowth phenotype associated with telangiectasias (Fig. 7f). However, the scRNA-seq was performed on tissues at the time of AVM formation (see Fig. 6), where smaller vessels are regressing as flow is diverted to the shunt. Taken together, our analysis strongly supports the idea that Diseased EC represent a remodeling population of cells^48^. For example, we note downregulation of genes associated with vessel stability, such as KLF2, FZD4, NOS3, and TSPAN12^48^ (Fig. 7f). Concomitantly, we see an upregulation of genes directly associated with vessel regression and pruning, including TAGLN2, CDKN1A, and MAPK10 ^48–50^ (Fig. 7g).

Several genes associated with matrix and matrix remodeling are also expressed in Diseased EC, including FN1, COL1A1, COL1A2, COL3A1, COL5A2, COL6A1, COL6A2, COL6A3, COL8A1, COL12A1, TGFBI, PCOLCE, VCAN, MMP2, TIMP1, TIMP3, SERPINE1, ADAMTS2, ADAMTS12, and ADAM12. Furthermore, we detected significant downregulation of basement membrane proteins COL4A2 and LAMA4, and significantly increased expression of LAMB1 (Supplementary Fig. 12f). Using immunofluorescence of Alk1-intact (no IPTG) vs. Alk1-deficient (IPTG-treated) EC in 2D monoculture, we validated this latter scRNASeq observation and found that at days 2 and 7, Alk1-deficient EC exhibit a significant increase in overall laminin production and deposition using a pan-laminin antibody (Supplementary Fig. 13a, b). Meanwhile, we detected no significant change in fibronectin expression at these same timepoints (Supplementary Fig. 13c, d). Our scRNASeq analysis enabled us to also examine effects of endothelial Alk1-deficiency on basement membrane protein expression of stromal cells. Comparison of stromal cell populations from Alk1-deficient networks (vs. Alk1-intact control networks) revealed significant changes in FN1 expression in fibroblasts and *SPARC* expression in smooth muscle cells of Alk1-deficient sh-*ACVRL1* networks (Supplementary Fig. 12g, h).

In our scRNASeq analysis, we also observed that both pro- and anti-angiogenic semaphorins are expressed, notably SEMA3A, SEMA3C and SEMA5A. PDGFB, which is involved in mural cell recruitment to vessels, was also reduced in Alk1-deficient Diseased ECs. Consistent with these data, gene ontology (GO) analysis of upregulated genes in Diseased EC showed an association with EC migration (Fig. 7h). We also examined expression of secreted cytokines and found significant increases in expression of *CXCL1* and *CCL2* in Diseased EC (Supplementary Fig. 12i). In contrast, GO analysis of upregulated genes in control networks showed an association with establishment of endothelial cell barrier, regulation of angiogenesis, and regulation of vasculature development (Fig. 7i). Finally, pathway analysis focusing specifically on the PDGF pathway highlighted the loss of PDGFB-PDGFRβ signaling from EC to pericytes and SMC (Fig. 7i). Thus, Diseased EC appear to represent a population of cells that are actively remodeling vessels from a stable and complex vascular network to a relatively small number of large AVM-like vessels.

### VEGF signaling inhibitors prevent but do not appear to regress lesions

**Fig. 8:**
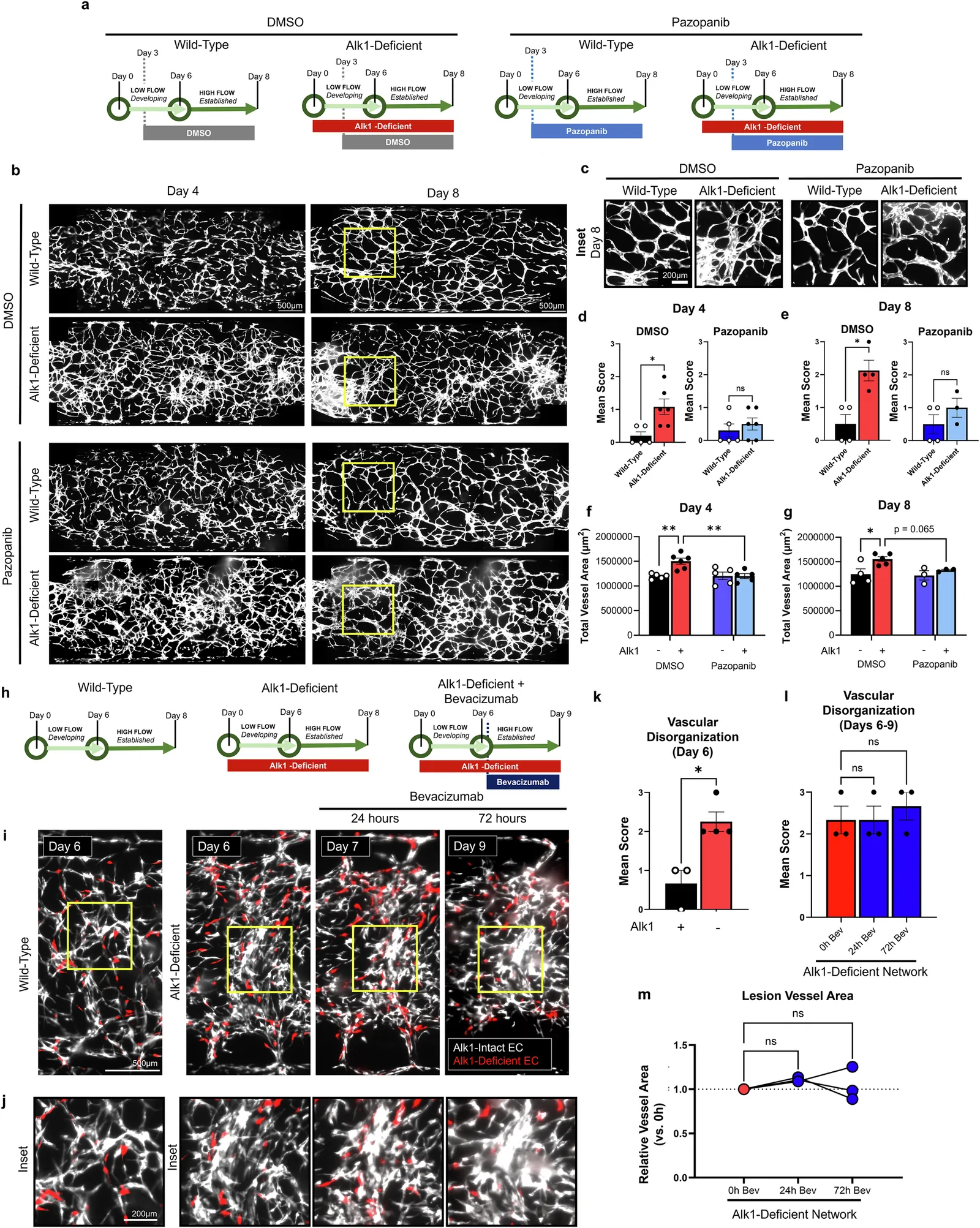
VEGF signaling inhibitors prevent but do not appear to regress microvascular lesions in HHT-VMO. **a** IPTG was administered to initiate Alk1 knockdown at day 0 and 100 nM Pazopanib (or DMSO) was added to circulating media beginning at day 3. **b** Pazopanib prevented subsequent appearance of microvascular lesions in IPTG-induced Alk1-knockdown networks. **c** Insets of (**b**), yellow boxes. **d**, **e** Vascular disorganization scores (Sample size: Day 4: DMSO/Wild-Type: *n* = 5, DMSO/Alk1-Deficient: *n* = 6, Pazopanib/Wild-Type: *n* = 5, Pazopanib/Alk1-Deficient: *n* = 6 independent biological replicates, Day 8: DMSO/Wild-Type: *n* = 4, DMSO/Alk1-Deficient: *n* = 4, Pazopanib/Wild-Type: *n* = 4, Pazopanib/Alk1-Deficient: *n* = 3 independent devices, Statistics: Mann–Whitney rank sum test, *p*-values: Day 4 – DMSO: *p* = 0.0216, Day 4 – Pazopanib: *p* = 0.5238, Day 8 – DMSO: *p* = 0.0286, Day 8 – Pazopanib: *p* = 0.3143). **f**, **g** Vessel area are elevated with IPTG-induced Alk1-knockdown in DMSO-treated vehicle control, but not in networks treated with Pazopanib at days 4 and 8. (Sample size: Day 4: DMSO/Wild-Type: *n *= 5, DMSO/Alk1-Deficient: *n *= 5, Pazopanib/Wild-Type: *n *= 6, Pazopanib/Alk1-Deficient: *n *= 5 independent devices, Day 8: DMSO/Wild-Type: *n *= 4, DMSO/Alk1-Deficient: *n *= 5, Pazopanib/Wild-Type: *n *= 3, Pazopanib/Alk1-Deficient: *n *= 3 independent devices, Statistics: Two-tailed 2-way ANOVA with mixed effects and adjustment for multiple comparisons, *p*-values: Day 4: DMSO – Wild-Type vs. Alk1-Deficient: *p *= 0.0012, Pazopanib – Wild-Type vs. Alk1-Deficient: *p *= 0.9582, Wild-Type – DMSO vs. Pazopanib: *p *= 0.9128, Alk1-Deficient – DMSO vs. Pazopanib: *p *= 0.0014, Day 8: DMSO – Wild-Type vs. Alk1-Deficient: *p *= 0.0118, Pazopanib – Wild-Type vs. Alk1-Deficient: *p *= 0.4078, Wild-Type – DMSO vs. Pazopanib: *p *= 0.8241, Alk1-Deficient – DMSO vs. Pazopanib: *p *= 0.0655). **h** IPTG was administered to initiate Alk1 knockdown at day 0, and 100 µg/mL bevacizumab was added to circulating media at day 6 after pronounced lesion structures were confirmed to have developed. **i** Bevacizumab was given for 72 h and changes in lesion appearance were assessed. **j** Insets of (**I**), yellow boxes. **k** The induction of lesions was confirmed at day 6 via vascular disorganization score (Sample size: Wild-Type: *n *= 3 independent devices, Alk1-Deficient: *n *= 4, Statistics: Mann–Whitney rank sum test, *p *= 0.0286. **l** Exposure to bevacizumab did not significantly alter vascular disorganization score (Sample size: *n *= 3 independent devices, Statistics: Paired Friedman test, *p *> 0.9999) or **m** overall lesion area (Sample size: *n *= 3 independent devices, Statistics: Ordinary one-way ANOVA with adjustment for multiple comparisons, *p*-values: 0 h vs 24 h: *p *= 0.4198, 0 h vs. 72 h: *p *= 0.8520). All plotted data are presented as mean values ± standard error.

A primary form of HHT disease management involves treatment with VEGF inhibitors Bevacizumab, a VEGF blocking antibody, is already widely used in HHT, several studies having shown it to be safe and effective in both limiting lesions and improving bleeding symptoms in patients^51–56^. Additionally, pazopanib, a receptor tyrosine kinase inhibitor with specificity for VEGFR, is also reported to improve HHT bleeding^57–59^, but it remains unknown how pazopanib may affect lesions mechanistically. To assess whether the formation of lesions in the HHT-VMO could be prevented by application of pazopanib, we seeded sh-*ACVRL1* EC into the HHT-VMO and added IPTG from day 0. We then added 25 nM (*data not shown*) or 100 nM pazopanib (or DMSO vehicle) to the perfusing medium beginning at day 3 (Fig. 8a), at which time our data have shown that Alk1 remains necessary to prevent vascular lesions (Fig. 4). Consistent with the clinical data showing bleeding and anemia improvement with clinical administration of pazopanib^57,59^, as well as reports that VEGF signal inhibition by bevacizumab reduces new lesion development in HHT^56,60^, we also observed an inhibition of lesion formation when pazopanib was added to IPTG-treated networks (Fig. 8a–g) with resultant vascular networks appearing significantly normalized at days 4 and 8 relative to vehicle- and IPTG-treated networks.

Next, we tested whether VEGF inhibition might induce regression of established lesions (Fig. 8h–j). Bevacizumab was selected for this study given its well-established use in the HHT patient population^51–56^, and case reports that bevacizumab improves lesion burden in HHT (e.g., reviewed in Arizmendez et al.^56^). We confirmed development of dilated microvascular lesions at day 6 in IPTG-treated devices (Fig. 8k), and then added 100 µg/mL bevacizumab into the perfusion medium for 72 h, mimicking the intravenous delivery used for HHT patients^51,52,56^. Perhaps surprisingly, bevacizumab failed to improve Alk1-deficient networks (Fig. 8l), and no change in lesion area was observed (Fig. 8m). Together, our data show that pharmacological VEGF inhibition prevents the appearance of lesions, but that VEGF inhibition may be ineffective in resolving established malformations.

## Discussion

Owing to their low-cost, reproducibility, and ease-of-use, OoaC/MPS technologies offer new avenues for the study of healthy physiology, disease biology, and preclinical drug screening using fully human tissues^27,29,61,62^. In particular, vessel-on-a-chip platforms are a potentially powerful tool for studying the pathogenesis of vascular malformation, especially in designs where EC are not constrained to form vessels along pre-established microfluidic channels and structures^29^. For example, in our base vascularized micro-organ (VMO) platform (as well as earlier prototypical iterations of this device), co-seeded human EC and stromal cells freely self-organize in response to interstitial flow to form a perfused microvascular network within a three-dimensional hydrogel microenvironment^30–33^. Recently, Soon and colleagues^63^ used a similar design to model brain arteriovenous malformations (AVM) by co-mixing in EC expressing mutant variants of *KRAS4*. Resulting networks were abnormal in appearance and included regions of dilated vessels. The current study underscores that vessel-on-a-chip technology that incorporates flow can serve as a basis for modeling vascular malformation defects relevant to several disease contexts, including HHT. Indeed, in the present work, we were able to show that under physiologic flow conditions Alk1-deficiency induces vascular malformations mirroring those seen in vivo. Moreover, our data suggest that only a small number of mutant cells are required in a vessel to trigger lesion formation, consistent with cell non-autonomous effects. Yet, even the abnormalities in EC size, proliferation, and extracellular matrix production we see in 2D monoculture are not sufficient for AVM formation in the base, low-flow VMO platform (i.e., Fig. 1)—additional signals are required.

Recently, Orlova and colleagues seeded HHT1 patient-derived human iPSC-derived (*ENG-*haploinsufficient) EC (iPSC-EC) into a vessel-on-a-chip platform. Resulting networks were distinct in morphology compared to microvessels formed from isogenic control iPSC-EC; surprisingly however, although microvessels were more permeable and fragile, HHT-mutant microvascular networks had reduced (rather than increased) mean vessel diameter and vascular density, suggesting that HHT-like vascular lesions did not develop in this setting. Two possible explanations can be proposed. The first is that a context-dependent signal necessary to drive HHT-associated vessel malformations (i.e., telangiectasias and shunts) may be absent in this vessel-on-a-chip model. The second is that the patient-derived iPSC-EC are heterozygous for HHT-causing gene mutations and retain residual gene expression from their remaining intact allele; as such, a second-hit LoH that ablates residual Alk1 or Eng gene expression—a proposed precursor to lesion formation in HHT patients^12,13^—may be required. Mounting evidence suggests this to be the case^13,43–45^.

We and others^23,39,64^ believe that an additional critical signal for AVM formation in HHT is hemodynamic flow. Using an HHT mouse model, Larrivee and colleagues showed that shunts typically form in high-flow regions of the neonatal mouse retina close to the optic nerve^39^, where in silico modeling predicts wall shear stress values are higher than in the retinal vascular periphery^41^, and where the retinal vasculature typically undergoes EC quiescence, arteriovenous specification, and vessel organization and maturation^65,66^. Genet et al. recently showed that cell cycle arrest is reduced in lesions that form in high-flow regions of the neonatal retina vasculature of HHT mouse models^67^, and Rochon and colleagues show in zebrafish models that flow-sensitive EC migration is dysregulated with Alk1-deficiency^23^; both findings may reflect a defect in mechanosensitive signaling transduction in mutant EC. In the current study, we find that gravity-driven flow in our base VMO platform produces shear stress values of ~0.5 dynes/cm^2^—clearly sufficient to support the formation of vessels-on-a-chip^31^, but apparently insufficient to support the development of AVMs (as evidenced by our finding that Alk1-deficient EC do not generate shunts in the base VMO, Fig. 1 and Supplementary Fig. 1).

To address these and other issues, we redesigned our existing VMO platform to support a significantly increased gravity-driven intravascular flow, resulting in ten-fold greater wall shear stress values of up to ~5 dynes/cm^2^. We also enlarged the tissue chamber to support a broader range of flow and shear stress profiles across the resulting microvascular network. The HHT-VMO design supports microvascular network formation similarly to the original VMO, and higher flow seems to support additional arteriovenous specification. Seeding of Alk1-deficient EC under lower flow conditions produces hyperdense microvessel networks with development of enlarged and dilated focal lesions reminiscent of HHT patient telangiectasias. Further enrichment for more penetrant Alk1-deficiency in EC drove the development of shunt-like AVM structures under higher flow conditions. Importantly, we demonstrate in several experiments that lesions respond similarly to in vivo HHT mouse models. For example, consistent with published work in mice^20,21^, Alk1-deficiency during periods of active vessel growth produces lesions in the HHT-VMO, whereas loss in established vessels does not. We also showed that treatment with the VEGFR inhibitor pazopanib—which appears to reduce HHT-associated bleeding in patients—prevented the appearance of lesions in Alk1-deficient networks. Meanwhile, VEGF signal blockade with 3 days of bevacizumab failed to meaningfully resolve pre-existing microvascular abnormalities, although it remains possible that longer-term bevacizumab might be more effective. Nonetheless, our results suggest that current disease management strategies that rely on VEGF antagonism may be more effective for preventing—rather than regressing—vascular lesions in HHT. While we cannot rule out the possibility that our findings are in part explained by the different mechanisms of VEGF inhibition between bevacizumab vs pazopanib, another explanation for our results may emerge from our scRNASeq analysis (Fig. 7) where we find that expression of both VEGF receptors—FLT1 (VEGFR1) and KDR (VEGFR2)—is downregulated in Diseased EC (the EC subpopulation that arise exclusively in IPTG-treated VMO-HHT networks and that specifically lack Alk1 transcript relative to all other EC) following appearance of lesion structures in Alk1-deficient networks. Given this, further inhibition of VEGF by bevacizumab would be predicted to be of limited effect on these cells, which might explain our observation that bevacizumab appears to be ineffectual at inducing lesion regression in our platform.

An important finding in this study is that lesions are comprised of both wild-type (i.e., Alk1-intact) and Alk1-deficient cells—seemingly randomly intermixed throughout the lesion although Alk1-deficient EC may be somewhat more abundant in lower-flow regions. Nonetheless, these studies highlight that only a small fraction of Alk1-deficient EC are needed to induce the appearance of a lesion, and that lesions arise as mosaic structures. This is in contrast to a model, with some appeal, whereby a clone of mutant cells expands to dominate the vessel wall, triggering lesion formation, as is reported to occur in Cerebral Cavernous Malformation^42^. Instead, our data favor a cell non-autonomous mechanism for shunt formation and are consistent with the recent work of Snellings and colleagues^13^, as well as deBose-Scarlett and colleagues^43,45^ in which HHT patient lesions are heterogeneous for both HHT gene-expressing EC and gene-ablated EC (by a proposed second-hit LoH event) with an estimated somatic mutant EC frequency of 8% or less across multiple tissues and HHT disease subtypes. These data are also consistent with mouse studies showing that minimal Endoglin deletion in a subpopulation of EC is sufficient to produce a lesion when combined with a pro-angiogenic stimulus^15^.

It is not yet clear how the presence of randomly positioned mutant EC can trigger a vessel-wide lesion, however our gene expression data hint that cell-cell communication may well play a major role as we noted mis-regulation of several junctional and signaling molecules in our single-cell RNA sequencing analysis (Fig. 7). Further studies are needed to address these ideas, including by taking advantage of the HHT-VMO’s ability to support real-time observations of lesion formation (as presented in Figs. 3, 5 and 6). We also see evidence in the scRNA-seq data for a population of Diseased EC that appear to be associated with genes related to vascular remodeling. During shunt formation in the HHT-VMO we see regression of smaller vessels concomitant with the growth of the shunt, which would be consistent with integration of the regressing cells into the larger vessel. Several genes known to be involved in vessel stability or pruning were differentially expressed in the Diseased EC cluster compared to other EC, including KLF2, FZD4, NOS3, TSPAN12, TAGLN2, CDKN1A, and MAPK10. Interestingly, PDFGB, which is a driver of mural cell recruitment to the vessel wall, was also reduced in knockdown cells leading to a loss of PDGFB-PDGFRβ (Fig. 7i). Lebrin and colleagues^68^ have previously shown that thalidomide treatment restores mural cell coverage in an animal model of HHT, at least in part through upregulation of PDGFB. Disrupted mural cell (pericyte and SMC) coverage in HHT is thought to relate to the fragile nature of the vessels and their propensity to bleed^69–71^. However, whether there was a loss of PDGFB expression in the mutant mice was not reported. Consistent with these data we also saw an increase in vessel permeability in our model (Fig. 5). Finally, the expression of multiple matrix and matrix remodeling genes, along with expression of both pro- and anti-angiogenic semaphorins, marks the Diseased EC cluster as a unique population of EC, driven by loss of BMP9-Alk1-mediated regulation, that seems to play a critical role in the dissolution of the vascular network and the formation of AVMs.

In summary, the HHT-VMO is an HHT-on-a-chip platform that reliably produces vascular lesions with morphological similarity to the telangiectasias and AVMs that arise in HHT patients. Importantly, our findings match those seen both in mouse models^10,19,21^ and in patient studies^4,7^. In addition, we have used the model to probe both the timing and the mechanism of lesion formation, and we find that AVM generation is a cell non-autonomous process. Thus, the HHT-VMO captures much of the physiology of intact tissue while offering the increased experimental reproducibility, scale, and ease-of-use benefits of in vitro models. Based on this work, we further adapted the HHT-VMO device for a standard microwell plate format, which allows us to integrate readouts that rely on equipment that are fitted for devices with a microplate footprint. Thus, we have significantly increased the scale of studies that can be supported by the HHT-VMO; importantly, mid-throughput drug discovery screens are certainly feasible. We therefore believe that the HHT-VMO can complement and augment existing in vivo models of HHT, and that together these tools will further propel research into HHT and the pathogenesis of vascular malformation.

## Methods

### Cells

HUVEC were selected as our generic EC type for this study because they are easy to isolate and culture, because they retain EC identity for multiple passages, and because we find that they reliably produce highly organized vascular networks in VMO platforms (e.g., Figs. 1 and 2) with a range of vessel calibers within the microvascular range (*data not shown*). We have previously also shown that HUVEC do not endogenously express high levels of arteriovenous specification markers, but are capable of acquiring expression of these specification markers in response to physiological flow^65^. For this study, primary human umbilical vein endothelial cells (HUVEC) were mechanically isolated^72^, and expanded in monolayer culture on gelatinized tissue culture flasks in either M199 medium (Thermo #11150067) supplemented with 10% fetal bovine serum (FBS) (Gibco #160000044), 50 µg/mL Endothelial Cell Growth Supplement (ECGS), (Corning #354006) and 50 µg/mL gentamicin (Thermo #15710064); or, in complete EGM-2 (Lonza CC-3162). Primary normal human lung fibroblasts (NHLF, Lonza CC-2512) were cultured in Dulbecco’s Modified Eagle Medium (Corning #10-017-CV) supplemented with 10% FBS (Gicbo) and 50 µg/mL gentamicin (Thermo). Cell lines were used regardless of cell sex, all cell lines were routinely tested to ensure they were negative for mycoplasma contamination, and primary cells were discarded after 8 passages in culture.

### siRNA

HUVEC were transfected with pooled si-*ACVRL1* (Ambion #4392420) or si-*ENG* (Dharmacon #L-011026-00) or non-targeting scrambled control (Dharmacon #D-001810-10 or Ambion #4390843) constructs, as previously described^65^. In brief, EC were cultured in complete EGM2 over-night prior to transfection (via Lipofectamine 2000, Thermo #11668019) with 8 nM siRNA for 24 h. EC were then washed with fresh complete EGM2 for an additional 24–48h prior to additional experiments.

### Fluorescent reporter and shRNA lentivirus

All lentivirus in this study was packaged with generation II lentiviral packaging vectors in 293T cells, and lentiviral-containing supernatant was tested for mycoplasma contamination and then concentrated in 50% polyethylene glycol (PEG) 8000 (Promega V3011) for at least 72 h at 4 °C. All lentiviral batches were then tested using HUVEC to calculate concentration of active lentiviral particles. For fluorescent reporter expression, HUVEC were transduced with lentivirus containing CMV promoter-driven red (mCherry, LegoC2), green (eGFP), or blue (Azurite) fluorescent reporters using 8–10 µg/mL polybrene (Fisher # NC9840454) at a multiplicity of infection (MOI) ≈ 2–5. This typically yields 80–90% transduction efficiency. To achieve inducible Alk1 knockdown, individual *ACVRL1*-targeting short hairpin-RNA (shRNA) clones (Table 1) were inserted into the MISSION® 3X-LacO Inducible shRNA plasmid backbone (Sigma). Both clones target a similar region in the *ACVRL1* transcript and produced similar levels of knockdown; thus, both were used interchangeably for this project. sh-*ACVRL1* was transduced into fluorescent EC at an MOI ≈ 0.8–1 in the presence of 8 µg/mL polybrene. Using a higher MOI compromised network formation in the VMO or HHT-VMO device, even in the absence of IPTG. Following transduction, EC were recovered from lentiviral transduction with fresh complete EGM2 for a minimum of 24 h, at which point they were seeded immediately into the VMO or HHT-VMO; or, for some studies, EC were enriched for high sh-*ACVRL1* transduction by being cultured for 48 h in complete EGM2 containing 1 µg/mL puromycin (Sigma #8833) and recovered in fresh complete EGM2 for an additional 48 h prior to co-seeding into the HHT-VMO device. To knockdown endogenous Alk1 expression, sh-*ACVRL1* expression was induced by addition of up to 3 mM IPTG (Sigma #16758) in static monoculture, or in circulating media of VMO or HHT-VMO devices.

### qPCR

Cells in monoculture were washed in PBS and flash-frozen at −80 °C or immediately processed for RNA. RNA was isolated in TRIZol™ reagent (Invitrogen) or using a column-based Quick-RNA microprep kit (Zymo R1051) according to manufacturers’ protocols. RNA concentration and purity was quantified using a Nanodrop 2000. cDNA libraries were generated using an iScript cDNA synthesis kit (Bio-Rad #1708890) according to manufacturer’s protocol. qPCR was performed in a Bio-Rad CFX96 machine using SYBR Green mastermix (VWR #101414) and qPCR primers listed in Table 1. All gene expression values were normalized to expression of 18S housekeeping gene.

### Immunofluorescence

Adherent cells were cultured in gelatinized Nunc Lab-Tek chamber slides (Thermo #177399) and fixed with 4% formaldehyde for 15 min. Following wash with PBS containing 1% BSA 0.25% Triton X, cells were blocked with PBS containing 3% BSA 0.25% Triton X for 1 h at room temperature. Cells were then incubated in primary antibody (Table 2) over-night at 4 °C. Following wash, cells were incubated in fluorescently-conjugated secondary antibodies (Invitrogen, 1:500) for 2 h at room temperature. Following wash in PBS, cells were incubated for 15 min in 10 µg/mL Hoechst 33342 diluted in PBS, washed in PBS, and up to 20 random fields were imaged on a Thermo Fisher EVOS M5000 inverted fluorescent microscope or a Leica SP5 confocal microscope. Mean fluorescence was quantified in ImageJ by subtracting mean fluorescence of background (measured in a 100px × 100px acellular region in the same field) from total mean fluorescence of each field, and normalizing against the number of cells in that field as quantified using a nuclear dye. For cell area, ImageJ was used to measure and calculate mean cell diameter and cell area of 25 randomly imaged cells in phase contrast. For nucleus area, ImageJ was used to calculate particle size for each Hoechst-positive nucleus captured in 5 randomly imaged fields. Hoechst-positive particles smaller than 100 pixels in area and/or less than 0.5 in circularity were excluded from the analysis.

**Table 2:** Primary antibodies for immunofluorescence

Antigen	Catalog number	Dilution
Alk1	Abcam #ab68703	1:100
αSMA	Sigma #A5228	1:300
CD31	Dako #M0823	1:500
Laminin	Invitrogen #PA1-16730	1:100
Fibronectin	Invitrogen #PA5-29578	1:100
eGFP	Invitrogen #CAB4211	1:100

### VMO and HHT-VMO devices

The vascularized micro-organ (VMO) microfluidic platform^30,31^ was fabricated and loaded as previously described, or adapted for improved study of vascular malformation in HHT (HHT-VMO). In brief, the VMO and HHT-VMO device features were etched onto a silicon wafer using ultraviolet-based photolithography, and then soft lithography techniques were used to generate a microfluidic feature layer in polydimethylsiloxane (PDMS) (Ellsworth, Dow Sylgard 184 #4019862). The features were then enclosed by plasma bonding (Harrick) to a thin commercial PDMS membrane (Pax). For VMO, the enclosed feature layer was plasma bonded (Harrick) to a (3-mercaptopropyl)trimethoxysilane-treated bottomless 96-well culture plate. For HHT-VMO, the feature layer was either PDMS stamped onto bottomless 2 mL cryovials (VWR #75852-324) or a custom 3D-printed reservoir layer produced out of high-temperature resin using a Formlabs 3D printer (Supplementary Fig. 7). Devices were then sterilized using either an autoclave or exposed to 20 min of ultraviolet light. Primary EC (less than passage 8) and NHLF (less than passage 10) were resuspended at cell densities of 8 × 10^6^ cells/mL for each cell type into a hydrogel comprised of 6.5 mg/mL fibrin (Sigma #341573) and supplemented with 0.2 mg/mL fibronectin (Sigma #F1141) and 10 µg/mL aprotinin (Sigma #A-6012). 1 mg/mL thrombin (Sigma T4648) was mixed into the hydrogel, and the hydrogel mixture was then immediately loaded into the VMO or HHT-VMO tissue chambers. Upper and lower microfluidic channels were then coated with extracellular matrix protein solution (0.5 mg/mL fibronectin, 0.5 mg/mL laminin (Fisher 23017015)) for 10 min before washout and establishment of gravity-driven complete EGM-2 media circulation through the device. Low interstitial flow (~2 dynes/cm^2^) of complete EGM-2 was applied such that it alternated direction across the tissue chamber every other day for 6 days, after which point unidirectional high flow (~10 dynes/cm^2^) was maintained for the remainder of the experiment. Vessel architecture was recorded every two days using an inverted epifluorescent microscope (Olympus or Zeiss). Vessel patency and intravascular flow was confirmed between days 6–8 by addition of sterile 50 µg/mL 70 kDa FITC- or rhodamine-conjugated dextran (Sigma #FD70S or #R9379) to the media reservoirs, and imaged in microvessel networks of the tissue chamber for up to 30 min. Fluorescently-conjugated dextran was then washed out by addition of fresh complete EGM2 to the media reservoirs.

### Vessel morphometry

VMO and HHT-VMO tissue chambers were imaged at 4× or 10× magnification every two days following initial device loading. For HHT-VMO, multiple images were taken across the tissue chamber, and either automatically or manually stitched using Fiji/ImageJ or Adobe Photoshop to capture the entire network. For morphometric analysis, networks were then cropped to a standard size (VMO: 800 ×1200 pixels; HHT-VMO: 1200 ×4000 pixels) to remove microfluidic channels and segmented using the Trainable Weka Segmentation plugin (FIJI/ImageJ)^73^. In some experiments, this process was automated using image analysis toolset developed in the Hughes lab^74^. Segmented images were then analyzed using the AngioTool plugin (FIJI/ImageJ)^75^ to obtain vessel area, vessel length, branchpoint number, and mean lacunarity values. For mean vessel diameter across the entire network, FIJI/ImageJ was used to automatically overlay a 10 pixel ×10 pixel grid overtop each segmented network image, identify those grid intersects that coincide with a microvessel, and to measure the corresponding vessel diameter at that point; any grid intersect that fell upon a vessel segment that had already been measured at an adjacent point was excluded from analysis so as not to oversample enlarged vessel structures. For mean vessel diameter across first order vessels, FIJI/ImageJ was used to divide first order vessel structures into top, middle, and bottom segments and to manually measure vessel diameter at evenly spaced intervals within each section. Those measurements were then averaged to obtain vessel diameter across the entire vessel length. For vessel disorganization score analysis, segmented image networks were de-identified using a randomly-assigned five-digit image code. Three investigators were trained using a training image set to assign codes based on morphological features of network architecture, as described in Supplementary Fig. 14. In brief, higher scores were associated with abnormal network appearance and development of vascular lesions. De-identified experimental image sets were then presented to at least two blinded investigators. In cases where scores from both investigators were highly variable from one another, investigators were re-presented with a de-identified image set that also included some training images interspersed within the dataset. If an investigator failed to reproduce their own scores from training, their scores were excluded from further analysis. Otherwise, investigator scores were averaged for each network once images were reidentified.

### In silico flow modeling

Image masks were generated as described above, and then were binarized, skeletonized, and traced using a custom MATLAB script. The traced images were converted to .DXF files using the DXFLib MATLAB package^76^ and imported into AutoCAD to overlay the schematics for the microfluidic devices. The traced vessels and microfluidic devices were imported into COMSOL 5.2.1, and the velocity and shear stress were calculated using the laminar flow steady-state model. Using the Bernoulli equation, all pressure heads were calculated based on medium height in the inlet/outlet wells and used as input to model the gravity-driven flow in the device. Further details on our workflow for in silico flow modeling, including relevant parameters and variables, are described in Hachey et al.^74^.

### Drugs

25 mg/mL stock bevacizumab was purchased through Tulane University’s Clinical Research Pharmacy and diluted directly into circulating media at a working concentration of 100 µg/mL to mimic the expected plasma concentration of bevacizumab when patients are given a dose of 5–7.5 mg/kg^51^. Pazopanib (MCE #HY-10208) was resuspended in DMSO at a 10 mg/mL stock concentration and added to the HHT-VMO cell culture medium at 100 nM concentration beginning on day 3. Pazopanib concentration was selected to approximate the range of doses that have been previously given to HHT patients to control bleeding symptoms (i.e., 50–400 mg per patient)^57–59^. Cell culture medium containing pazopanib (or DMSO only) or bevacizumab was refreshed every two days.

### Single-cell RNA sequencing sample preparation

The thin transparent membrane was carefully removed from the bottom of the PDMS layer to expose the tissue chambers of 12-device units. The chambers were washed with HBSS prior to adding digestion buffer (complete EGM2 supplemented with 400 U/mL nattokinase and 1 mg/mL collagenase type I) in a drop-wise manner. After incubation at 37 °C for 5 min and gentle mechanical dissociation with a micropipette, cell suspensions were collected and centrifuged (340 × *g* for 3 min). Cell pellets were resuspended in complete EGM2 at 1000 cells/mL and viability was checked using Trypan Blue to confirm >95% viability. The cellular suspensions were then loaded onto a Chromium Single Cell Instrument (10× Genomics) and processed to generate cDNA using 10× Genomics v2 chemistry according to the Chromium Single Cell 3′ Reagents kits v2 user guide^35^. After undergoing quantification and quality control of cDNA libraries using the Qubit dsDNA HS Assay kit (Life Technologies Q32851), high-sensitivity DNA chips (Agilent 5067-4626), and KAPA qPCR (Kapa Biosystems KK4824), cDNA were sequenced on an Illumina HiSeq4000 at UCI’s Genomics Research and Technology Hub to achieve an average of 50,000 reads per cell.

### Single-cell RNA sequencing alignment and processing

FASTQ files were aligned to GRCh38 and converted to a count matrix with the 10× Genomics CellRanger v6.0.0^77^ using the 10× Genomics Cloud Analysis. The filtered count matrices were loaded into RStudio (R version 4.0.4)^78^ using the Seurat::Read10X_h5 function and converted to a Seurat object using the Seurat R package (Seurat_4.0.0)^79^ with the Seurat::CreateSeuratObject function. Cells were removed if they contained greater than 15% mitochondrial RNA or had fewer than 200 features. In addition, a subset of ribosomal and mitochondrial RNA was removed to improve clustering. Data were normalized and log-transformed using the Seurat::NormalizeData and Seurat::FindVariableFeatures(nfeatures=2000, selection.method = “vst”) functions. The influence of the cell cycle between cycling and non-cycling cells was minimized by regressing the difference between the expression of cells in the S phase and G2/M phase using the Seurat::ScaleData function. The data was also scaled to create data in the SCT data slot of a Seurat object using the Seurat::SCTransform function. Doublets were removed using DoubletFinder_2.0.3^80^. The optimal number of dimensions was determined using the Seurat::ElbowPlot function. The data was simplified to two dimensions using the Seurat::RunUMAP function, and the nearest neighbors were found with the Seurat::FindNeighbors function. The resolution that drives cluster grouping was determined by using the clustree::clustree function^81^ to assess the stability of the clusters, as well as looking at the uniqueness of the differentially expressed genes. The cell types were determined using the SingleR^82^ package and limiting the database to only include cells closely related to those in the devices (endothelial cells, fibroblasts, pericytes, and smooth muscle cells). The control dataset contained 11,224 cells with 1289 endothelial cells (ECs), 8739 fibroblasts, 136 pericytes, and 1060 smooth muscle cells (SMCs). The sh-*ACVRL1* dataset contained 8541 cells with 1197 ECs, 6185 fibroblasts, 195 pericytes, and 964 SMCs.

### Single-cell RNA sequencing dataset integration and subsetting

Datasets were integrated using the scMC^83^ R package, which creates a shared reduced-dimensional embedding of cells corrected for technical variation, with the input being the “RNA” slot from the already processed individual datasets. Numerous dimensions were evaluated to test the stability of each. The labeled cell types determined the clustering. As flagged by SingleR^82^, the ECs for each dataset were subset and then integrated using scMC. The standard Seurat workflow was run to generate unsupervised clusters. Finally, the proportions of the datasets in each cluster were determined using the scMC::computeProportion function.

### Single cell RNA sequencing, differentially expressed genes, pathway analysis, and cell-cell communication

Differentially expressed genes (DEGs) for the Diseased EC population compared to all of the other EC populations were determined using the Seurat::FindMarkers() function with the DESeq2 technique. The DEGs were filtered by log fold change and percent expression. The top 30 genes were used for pathway analysis. The pathway analysis was conducted using the EnrichR^84^ package with the GO Biological Processes 2021 dataset^85,86^. Significant pathways related to endothelial cell function were highlighted in the dot plot. To compute cell-cell communication the EC labels were mapped onto the larger dataset. The EC were grouped into Diseased EC and the conglomerate of Control EC and the interactions with the surrounding stroma were determined using CellChat^87,88^ (Supplementary Fig. 12e).

### Statistics, rigor, and reproducibility

Power analysis was used to select the sample sizes needed to detect morphological morphometric differences in VMO or HHT-VMO experiments at power 0.8. Significant differences were detected using two-tailed parametric or non-parametric tests as indicated for each study. In all cases, adjustments were made for multiple comparisons and alpha was set at 0.05. In chip studies, studies were conducted on multiple independent VMO or VMO-HHT devices and using multiple primary cell lines.

### Ethics

This study does not involve experiments involving animals, human participants, or clinical samples.

### Reporting summary

Further information on research design is available in the Nature Portfolio Reporting Summary linked to this article.

## Supplementary information

Supplementary Information

Description of additional Supplementary files

Supplementary Data 1

Reporting Summary

Transparent Peer Review file

## Data Availability

All data supporting the findings of this study are available within the article and its supplementary files. Any additional requests for information can be directed to, and will be fulfilled by, the corresponding authors. Source data are provided with this paper. As feasible, analyzed data from this study will be made available through the Biosystics database (https://biosystics-ap.com/), which was formerly known as the Microphysiological Systems Database created by the University of Pennsylvania^89^ and the Gene Expression Omnibus (https://www.ncbi.nlm.nih.gov/geo/) (Accession #: GSE252666). All plotted data are also available for download through the Source Data table included with this manuscript.
