# Supplementary material for: A microphysiological system HHT-on-a-chip platform recapitulates patient vascular lesions
Source: Nat Commun. 2026 Aug 13;17:9719. doi: 10.1038/s41467-026-76448-w (PMC13562586; doi:10.1038/s41467-026-76448-w)
Supplement: Supplementary file 1 [file 41467_2026_76448_MOESM1_ESM.pdf]

SUPPLEMENTARY FIGURES

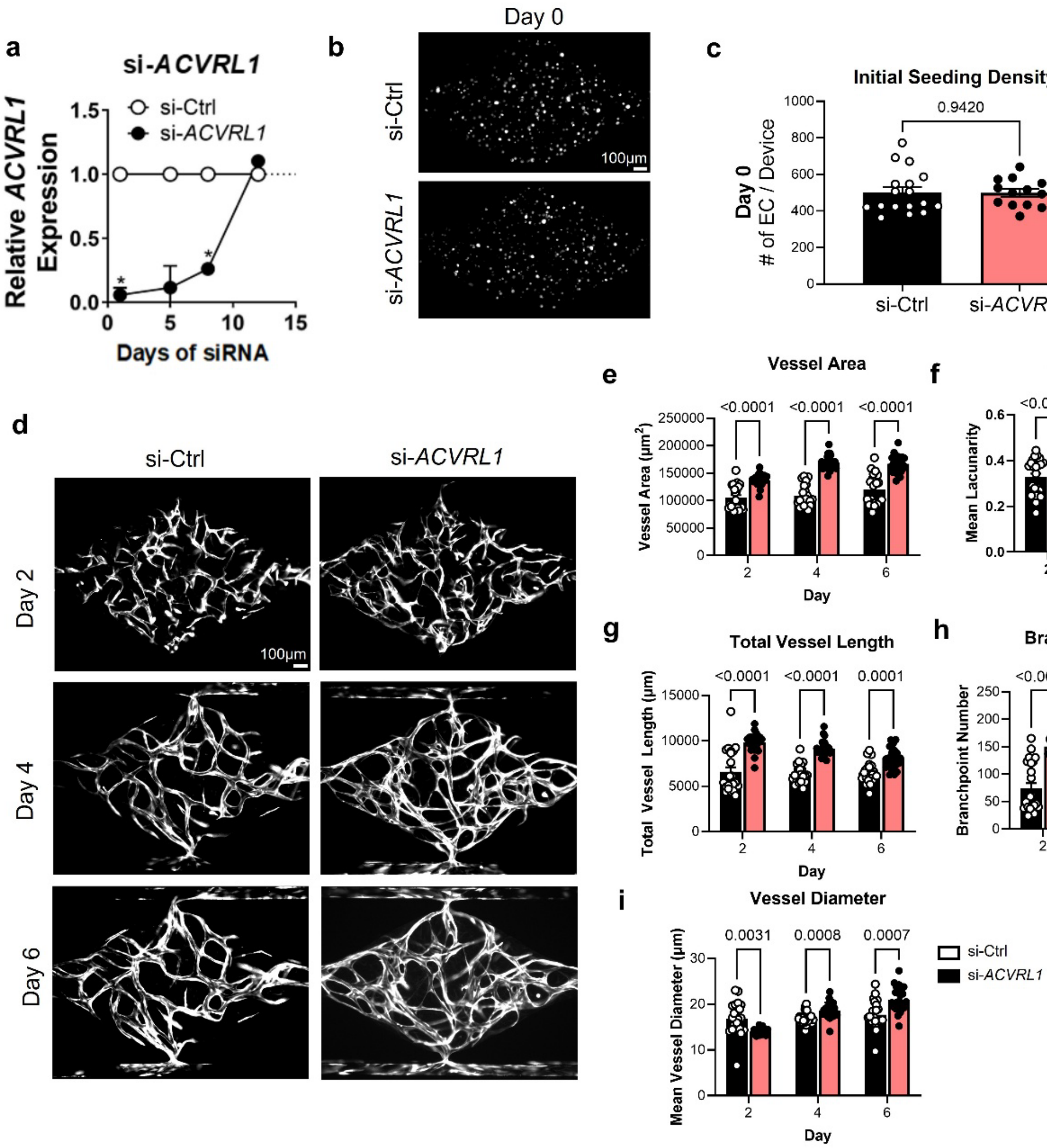

Supplementary Figure 1: Alk1 knockdown by siRNA induces formation of disorganized microvessels in VMO. a) si-*ACVRL1* knockdown of endogenous Alk1 is stable for 7 days but recovers by day 12. **b)** Initial seeding of si-Ctrl or si-*ACVRL1* into the VMO platform is **c)** equal across both conditions (Sample size: n=13-17 independent devices per group, Statistics: Unpaired Students' t-Test, ns – p > 0.05). **d)** VMO networks formed of si-*ACVRL1* EC appear more dense, as quantified by **e)** significantly increased vessel area, **f)** significantly decreased mean lacunarity, **g)** significantly increased total vessel length and **h)** branchpoint number at most timepoints, and **i)** significantly dilated vessels at days 4 and 6. (Sample size: n=13-16 independent devices per group, Statistics: Two-tailed 2-way ANOVA with mixed effects and adjustment for multiple comparisons). All plotted data are presented as mean values ± standard error.

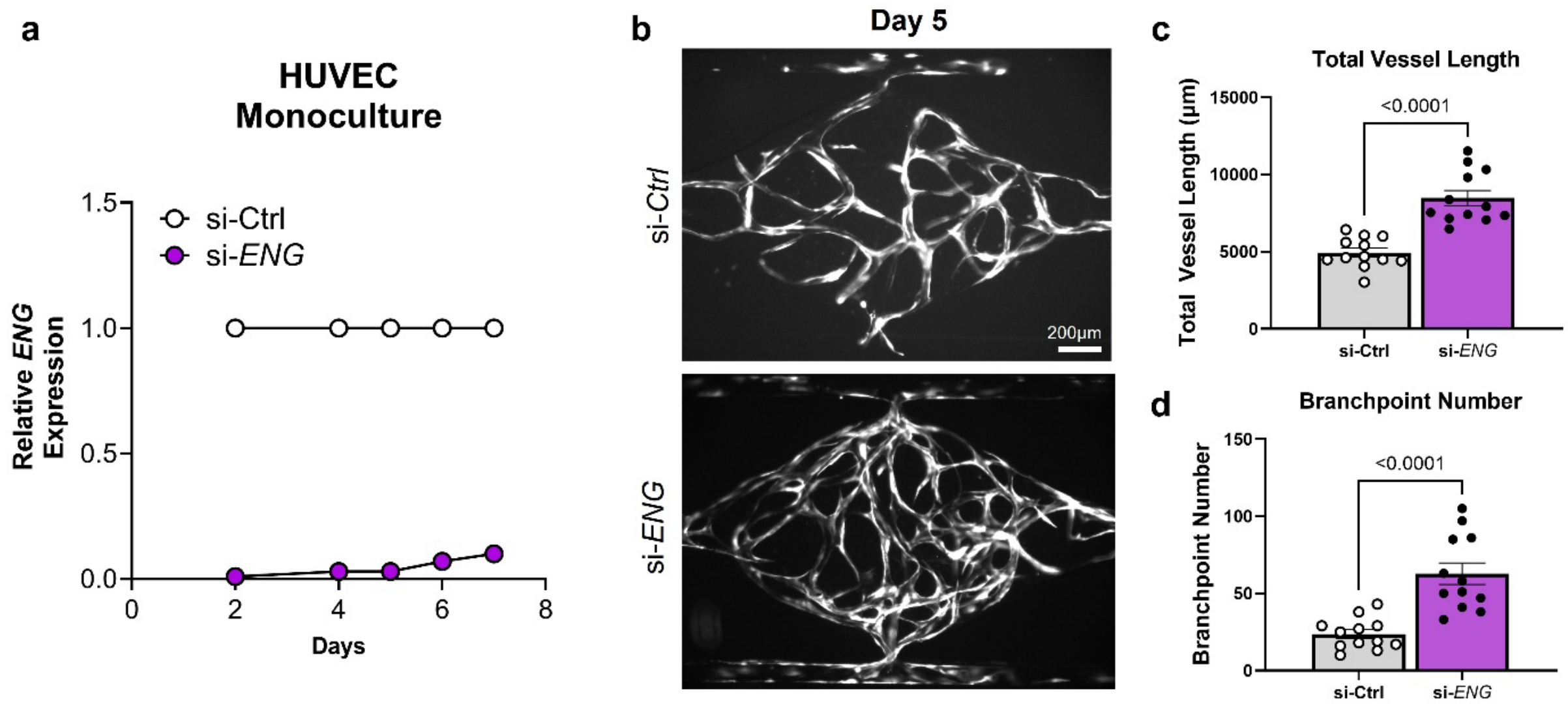

Supplementary Figure 2: *ENG* knockdown in EC also induces microvascular. a) siRNA targeting *ENG* (Eng) also induces knockdown of Eng in EC. **b)** si-*ENG* EC form hyperdense microvasculature in the VMO at Day 5, associated with increased **c)** total vessel length and **d)** branchpoint number. (Sample size: n=12 independent devices per group, Statistics: Unpaired Students' t-test). All plotted data are presented as mean values ± standard error.

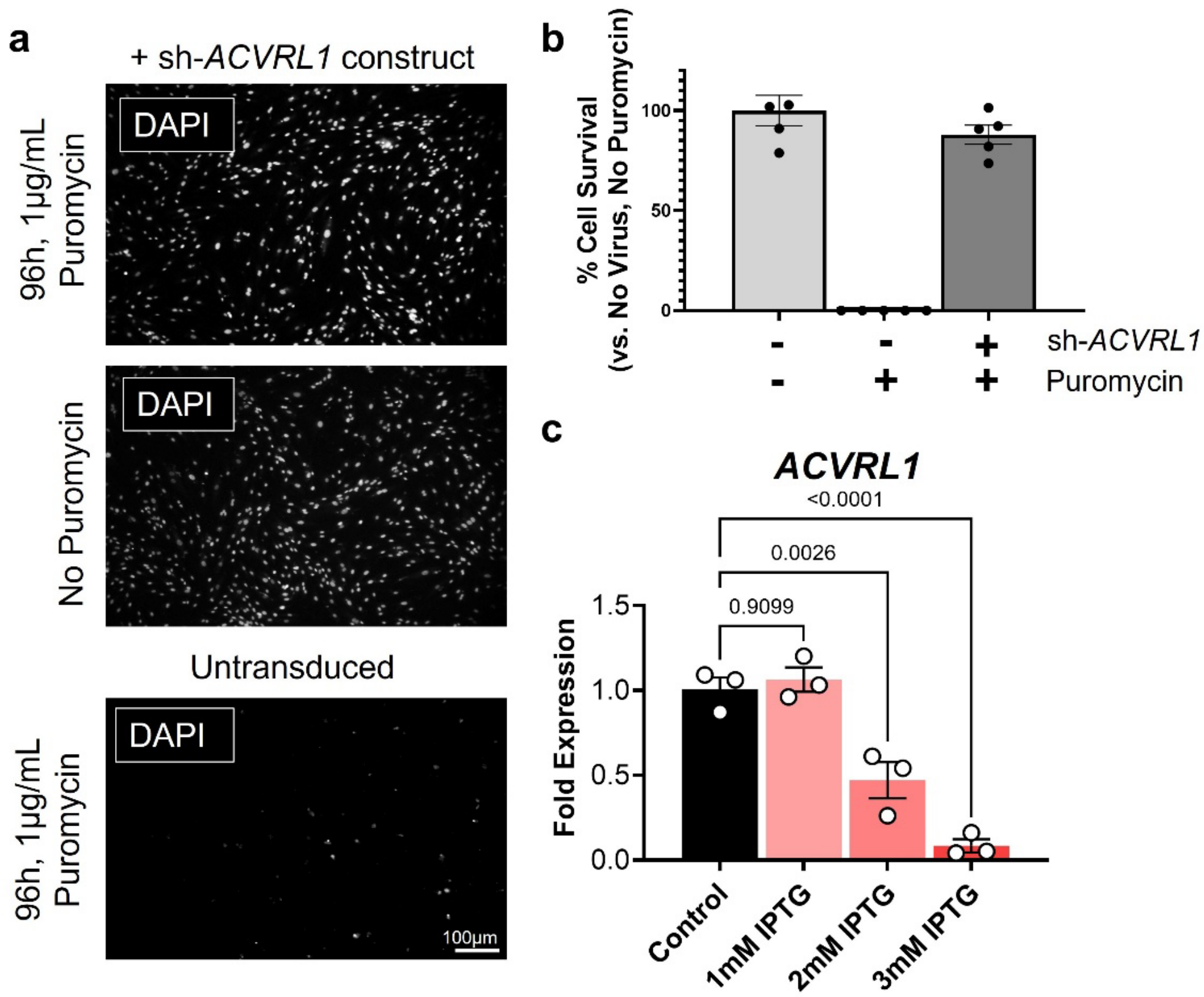

Supplementary Figure 3: Lentiviral transduction efficiency for sh-ACVRL1 construct. a) Transduction of primary EC with *sh-ACVRL1* construct renders cells resistant to puromycin whereas untransduced cells fail to survive puromycin exposure. **b)** >85% of EC survive 1ug/mL puromycin following transduction with sh-ACVRL1 construct (MOI≈1) indicating strong transduction efficiency. **c)** Primary EC transduced with sh-*ACVRL1* exhibit IPTG dose-dependent knockdown of endogenous *ACVRL1* (Alk1) transcript following 48h exposure to the inducing agent. (Sample Size: n=3 technical replicates per group, Statistics: Ordinary 1-way ANOVA with adjustment for multiple comparisons). All plotted data are presented as mean values ± standard error.

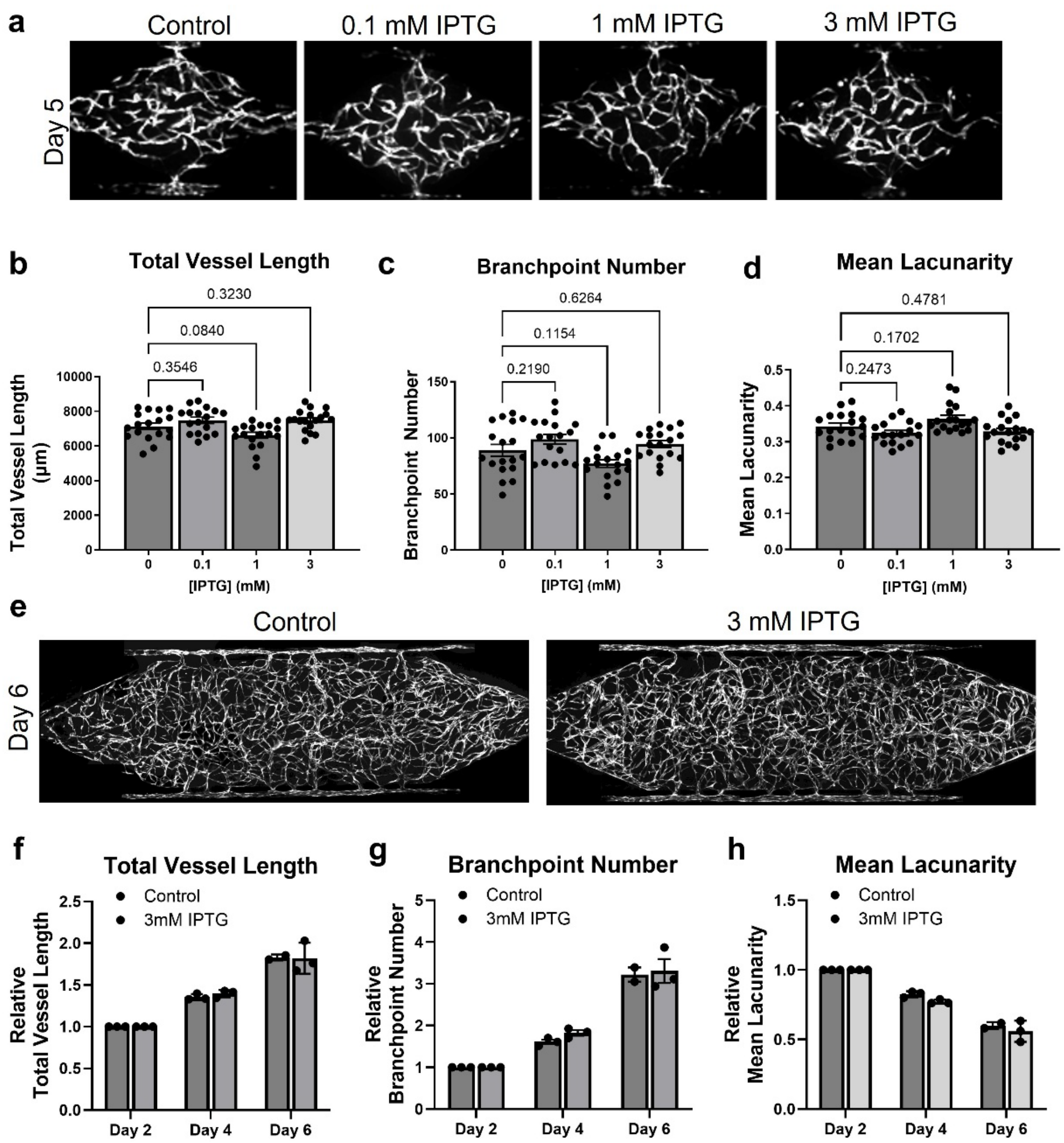

Supplementary Figure 4: IPTG does not alter network organization in VMO or VMO-HHT. a) 5 days of 0.1mM - 3mM IPTG to circulating media does not alter network organization for untransduced EC in the VMO, quantified as no change in **b)** total vessel length, **c)** branchpoint number, or **d)** mean lacunarity. (Sample size: Control: n=18, 0.1mM: n=17, 1mM: n=18, 3mM: n=18 independent devices for each treatment, Statistics: Two-tailed 1-way ANOVA with adjustment for multiple comparisons). **e)** 6 days of 3mM IPTG in circulating media of networks formed from untransduced EC in the VMO-HHT also does not alter network organization, quantified as no change in **f)** total vessel length, **g)** branchpoint number, or **h)** mean lacunarity (Sample size: Day 2-4: n=3 per group, Day 6: Control: n=2, IPTG: n=3 independent devices). All plotted data are presented as mean values ± standard error.

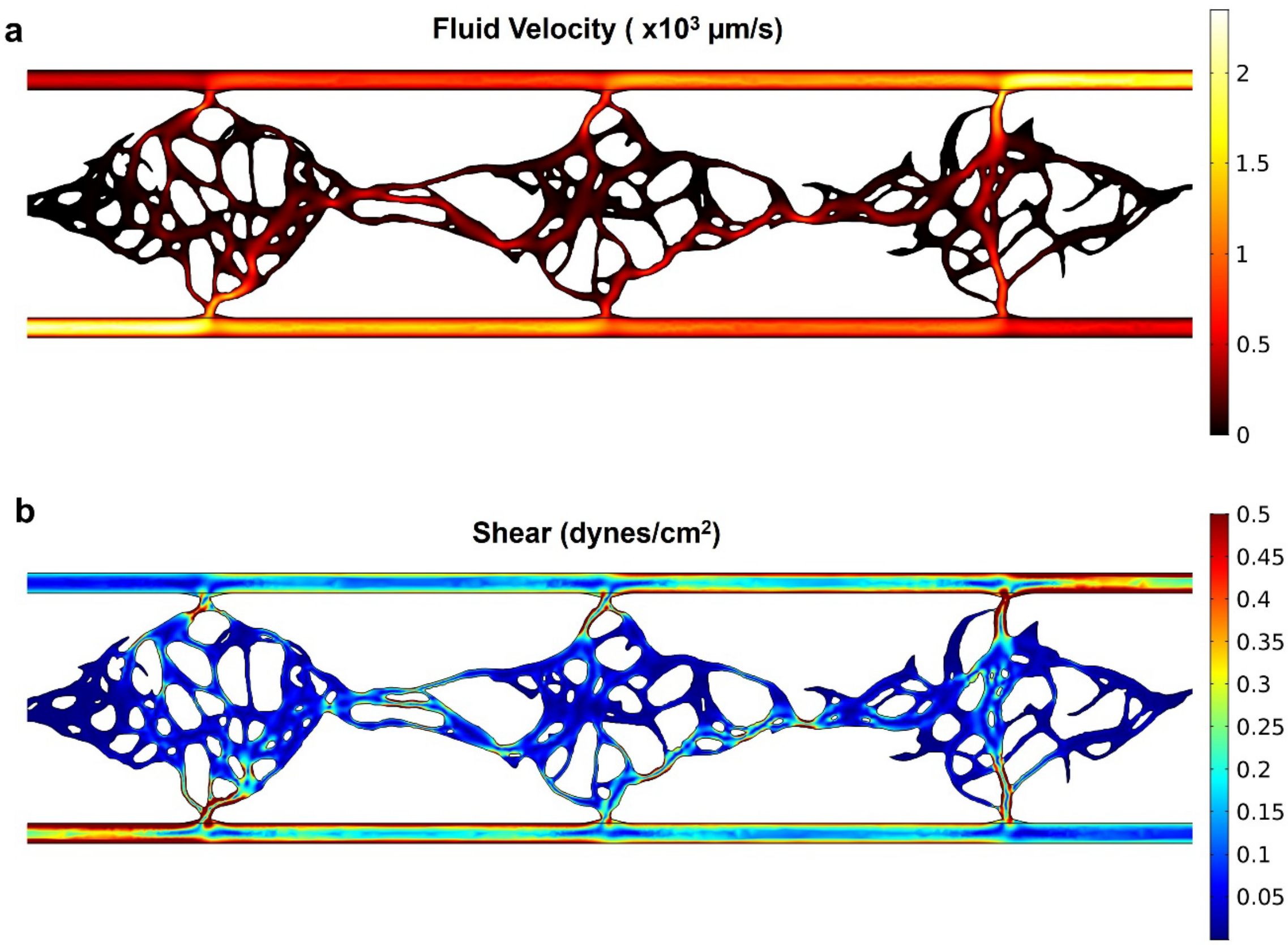

Supplementary Figure 5: *In silico* modeling of fluid velocity and shear stress in the VMO. COMSOL modeling of **a)** fluid velocity and **b)** fluid shear stress in VMO networks.

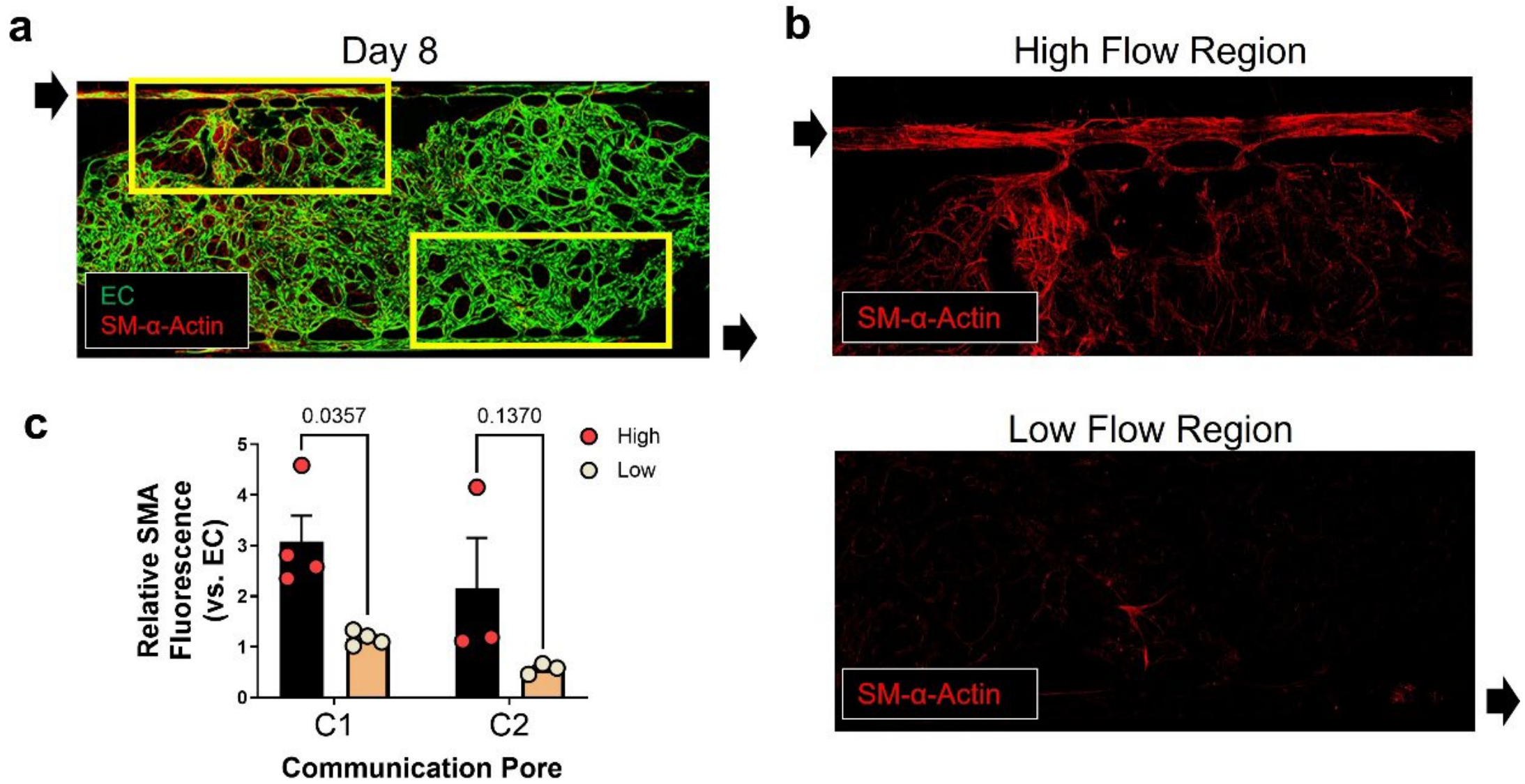

Supplementary Figure 6: Perivascular cell acquisition of SM-α-actin (SMA) expression in high flow regions. a) Exposure of an established microvascular network to physiological high flow for 48h **b)** induces expression of SMA (red) in high flow (but not low flow) regions. **c)** Relative SMA fluorescence (normalized to EC signal) was significantly increased at the first communication pore (C1) of the high flow region (relative to C1 in the low flow region). (Sample size: C1: n=4, C2: n=3 independent devices, Statistics: Two-tailed 2-way ANOVA with mixed effects and adjustment for multiple comparisons). All plotted data are presented as mean values ± standard error.

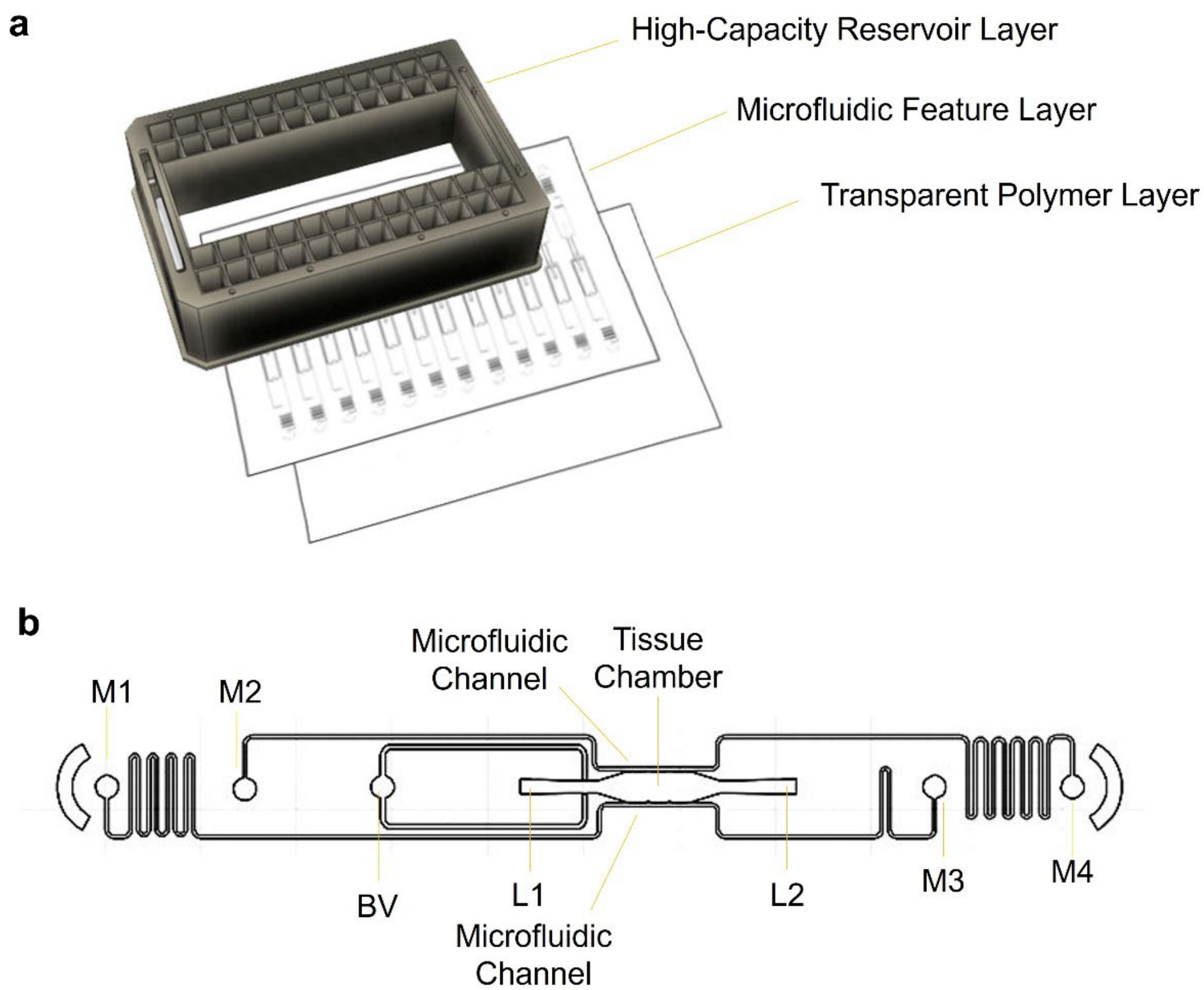

Supplementary Figure 7: Microplate-adapted versions of the VMO-HHT microfluidic platform. a) Microplate-adapted VMO-HHT was configured to fit a standard 96-well plate format. It is comprised of a 3D-printed high-capacity reservoir layer that fits a standard microplate lid, a middle microfluidic feature layer, and a transparent polymer layer. **b)** Each feature layer fits 12 microfluidic bioreactors that consist of a central tissue chamber lined by upper and lower microfluidic channels. The chamber is loaded through loading ports (L1, L2), and aided by the presence of a burst valve (BV). Microfluidic channels are connected to media inlets and outlets (M1 – M4) which circulate media contained in high-capacity reservoir wells. Device schematics created using AutoCAD.

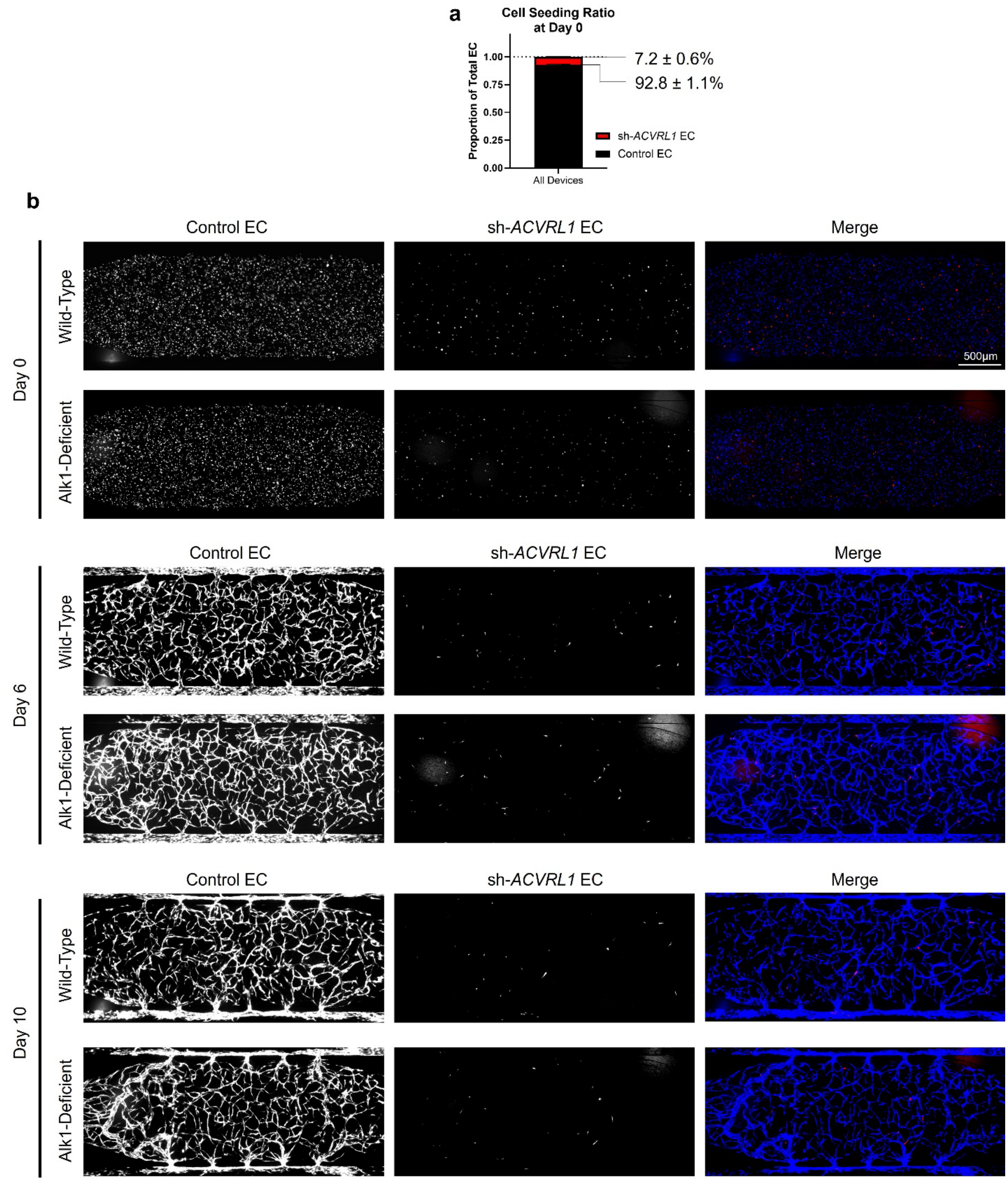

Supplementary Figure 8: Small subpopulation of Alk1-Deficient EC is sufficient to produce a lesion. a) Seeding of sh-*Alk1* EC at a proportion of 1:5 relative to control Alk1-intact EC is **b)** sufficient to produce a lesion by day 10 in Alk1-deficient networks. All plotted data are presented as mean values ± standard error.

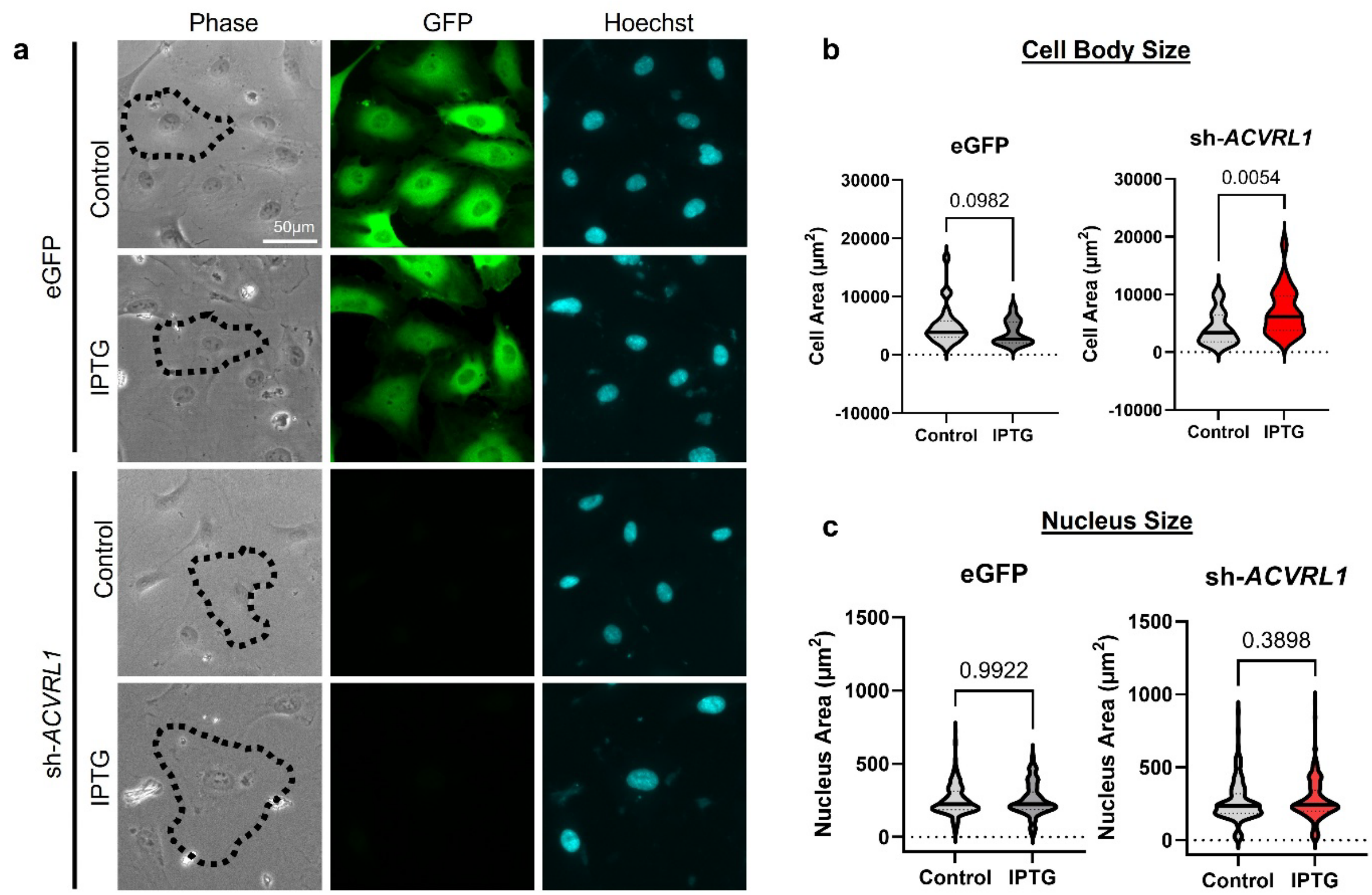

Supplementary Figure 9: Alk1-deficiency increases endothelial cell area. a) Primary HUVEC transduced with either sh-*ACVRL1* or control eGFP lentiviral plasmid (MOI ≈ 1) were exposed for 48h to media containing 3mM IPTG (or no IPTG control) prior to fixation and nuclear staining with Hoechst. GFP is present in all eGFP-transduced control EC confirming efficient lentiviral transduction and expression. **b)** IPTG treatment increased cell area in sh-*ACVRL1* (Alk1-deficient) EC, but not in control (Alk1-intact eGFP) EC. (Sample size: n=25 cells per group, Statistics: Unpaired Students' t-test). **c)** Although nuclei appeared larger in IPTG-treated *sh-ACVRL1* EC, we did not detect significant differences in nucleus area upon quantification (Sample size: eGFP / Control: n=294 nuclei, eGFP / IPTG: n=349, sh-*ACVRL1* / Control: n=195 nuclei, sh-*ACVRL1* / IPTG: n=238 nuclei, Statistics: Unpaired Students' t-test). All plotted data are presented as mean values ± standard error.

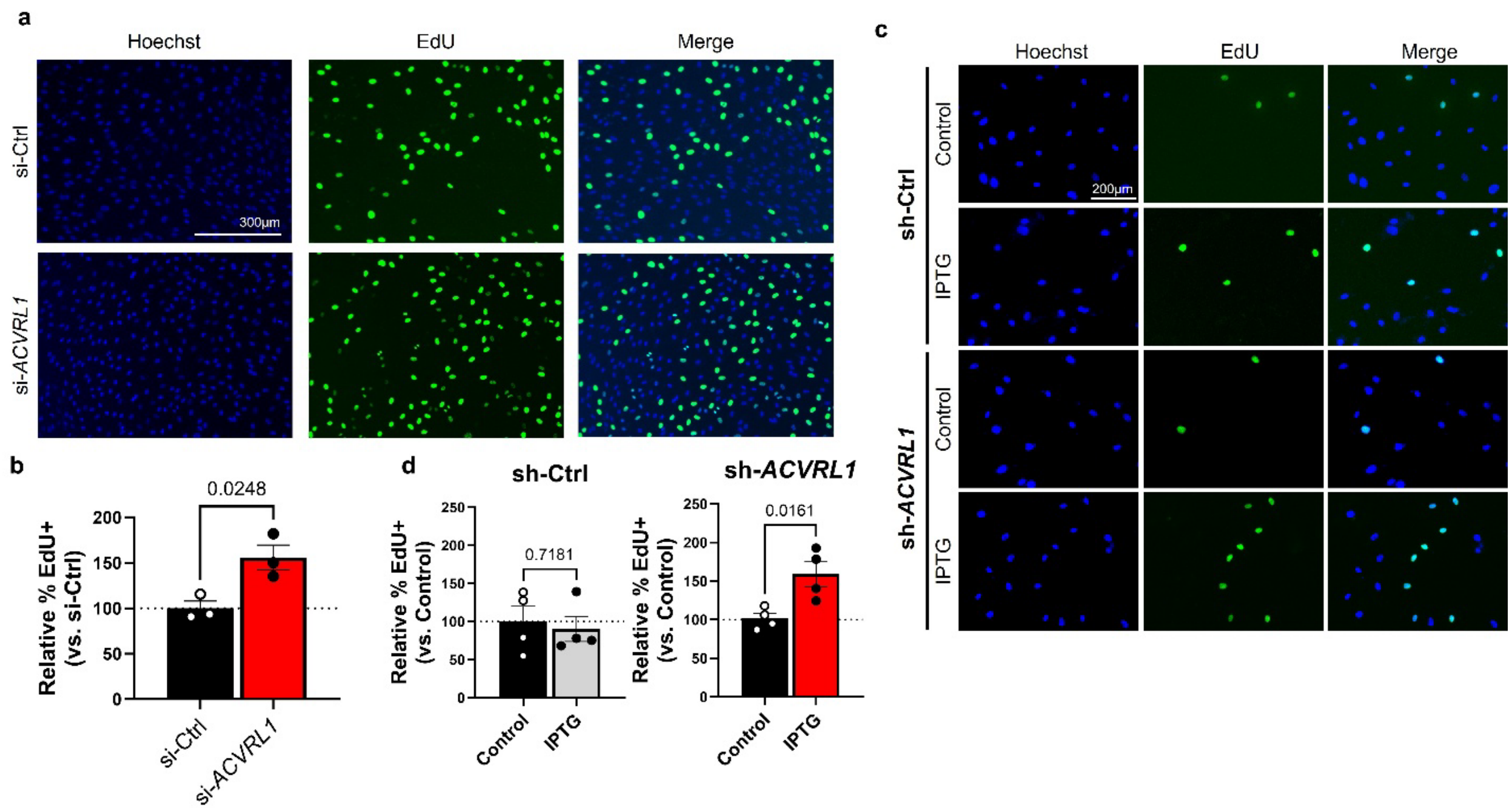

Supplementary Figure 10: Alk1-deficiency increases endothelial cell proliferation rate. 48-hour knockdown of endogenous Alk1 via **a-b)** silencing RNA (Sample size: n=3 independent cultures per group, Statistics: Unpaired Students' t-test) or **c-d)** IPTG-induced expression of sh-ACVRL1 significantly increases endothelial cell proliferation vs. si-Ctrl or Alk1-intact (i.e., no IPTG) *sh-ACVRL1* control cells, as measured by incorporation of the proliferation marker, EdU. There was no effect of IPTG on the proliferation of sh-Ctrl (Alk1-intact) endothelial cells (Sample size: n=4 independent cultures per group, Statistics: Unpaired Students' t-test). All plotted data are presented as mean values ± standard error.

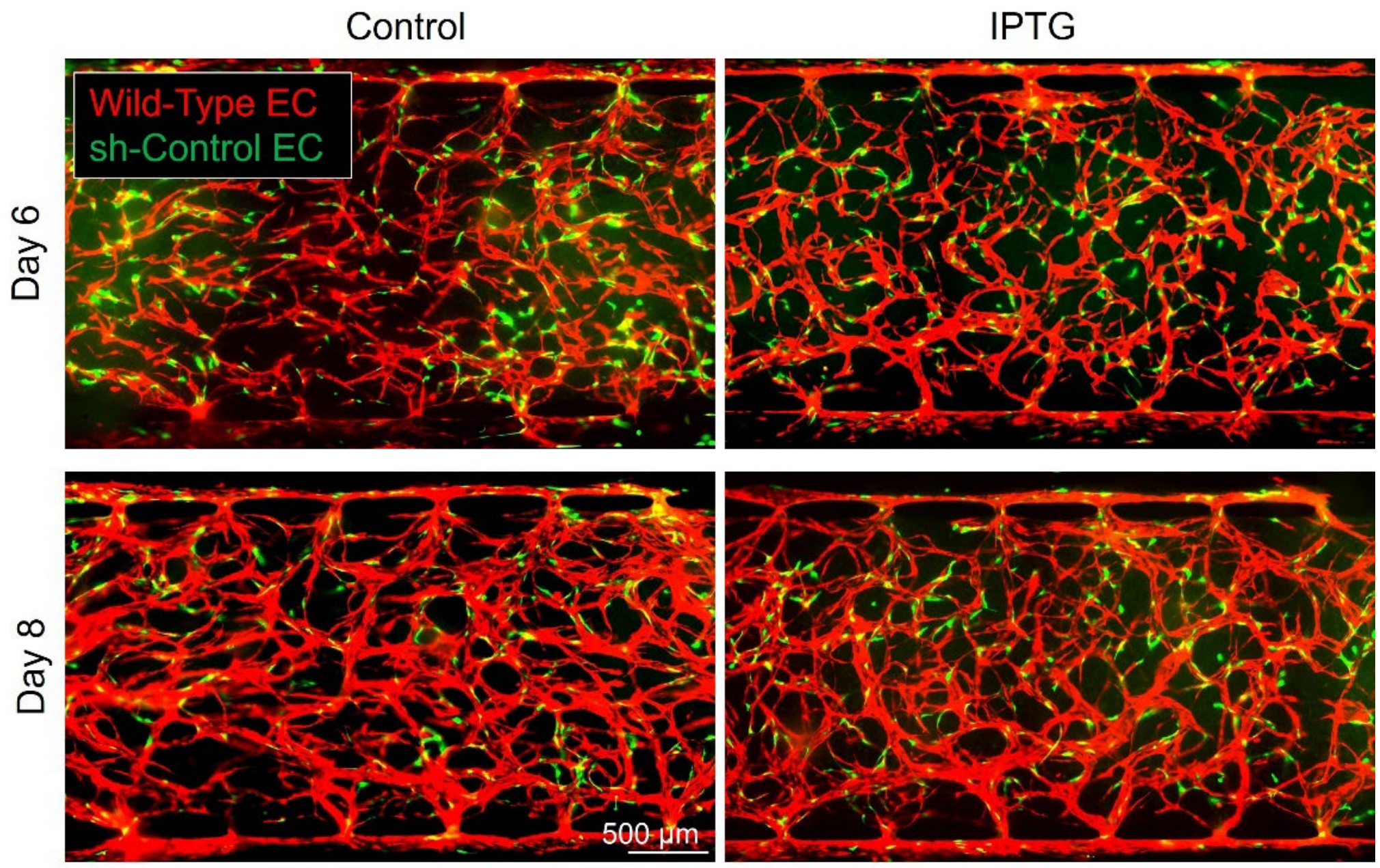

Supplementary Figure 11: sh-Ctrl does not alter network organization in VMO-HHT. EC transduced with IPTG-sensitive control vector (green) were co-mixed with wild-type (untransduced) EC (red) and seeded into the VMO-HHT. No vascular lesion phenotype was evident by day 6 or day 8 following, either in control or IPTG conditions.

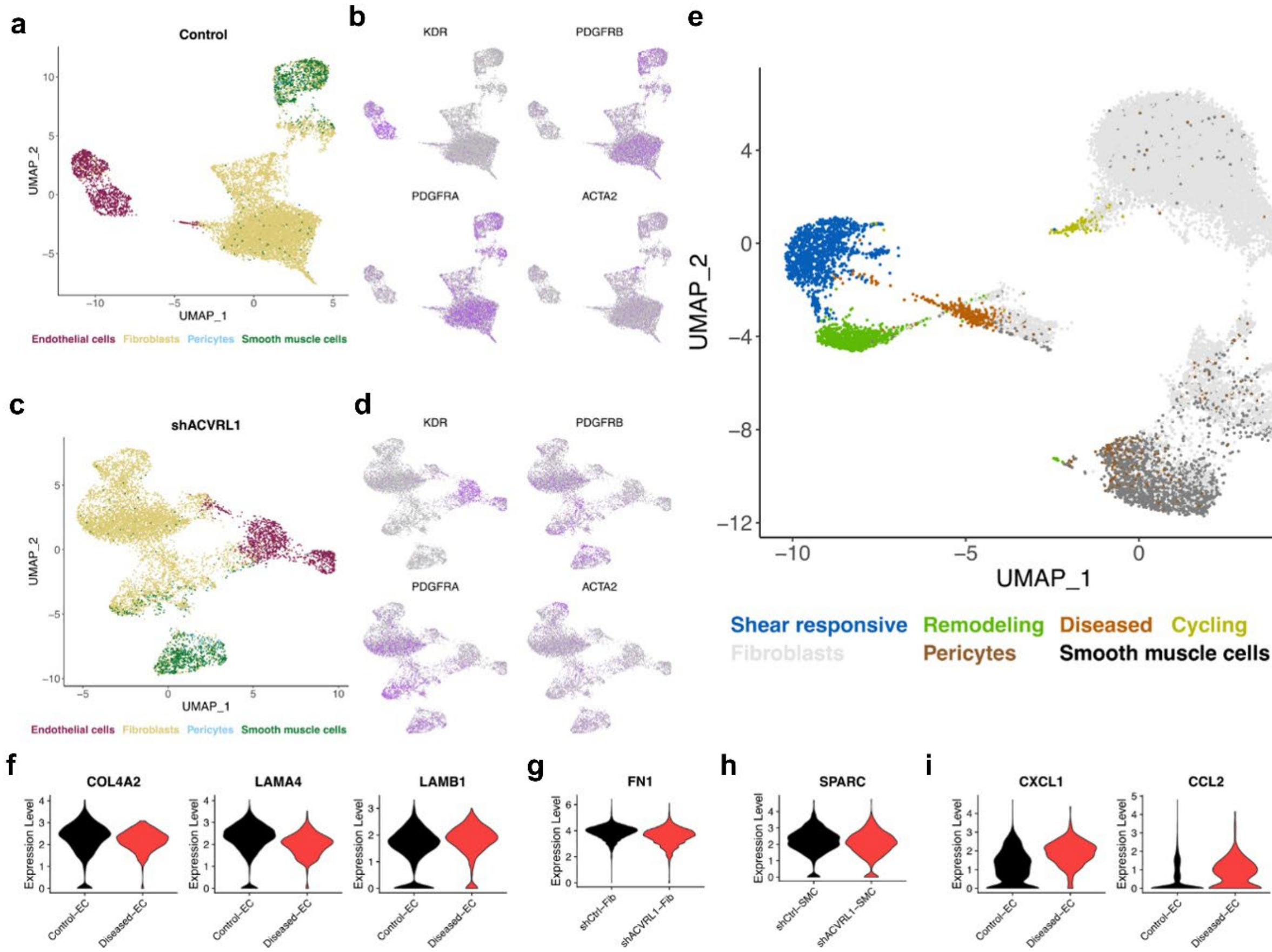

Supplementary Figure 12. Single-cell RNA sequencing. A-D) Single-cell RNA sequencing of the **a-b)** control dataset and **c-d)** shACVRL1 dataset showing that each are comprised of **B,D)** endothelial cells, fibroblasts pericytes, and smooth muscles with expression of marker genes shown. **e)** Integrated datasets with labeling for cell-cell communication. EC are labeled from assignment based in Figure 7c. Violin plots comparing expression between **f)** Control vs Diseased EC for basement membrane proteins, and **g)** shCtrl vs shAVRL1 for fibroblast and **h)** smooth muscle cell basement membrane proteins, and **i)** Control vs. Diseased EC for cytokines.

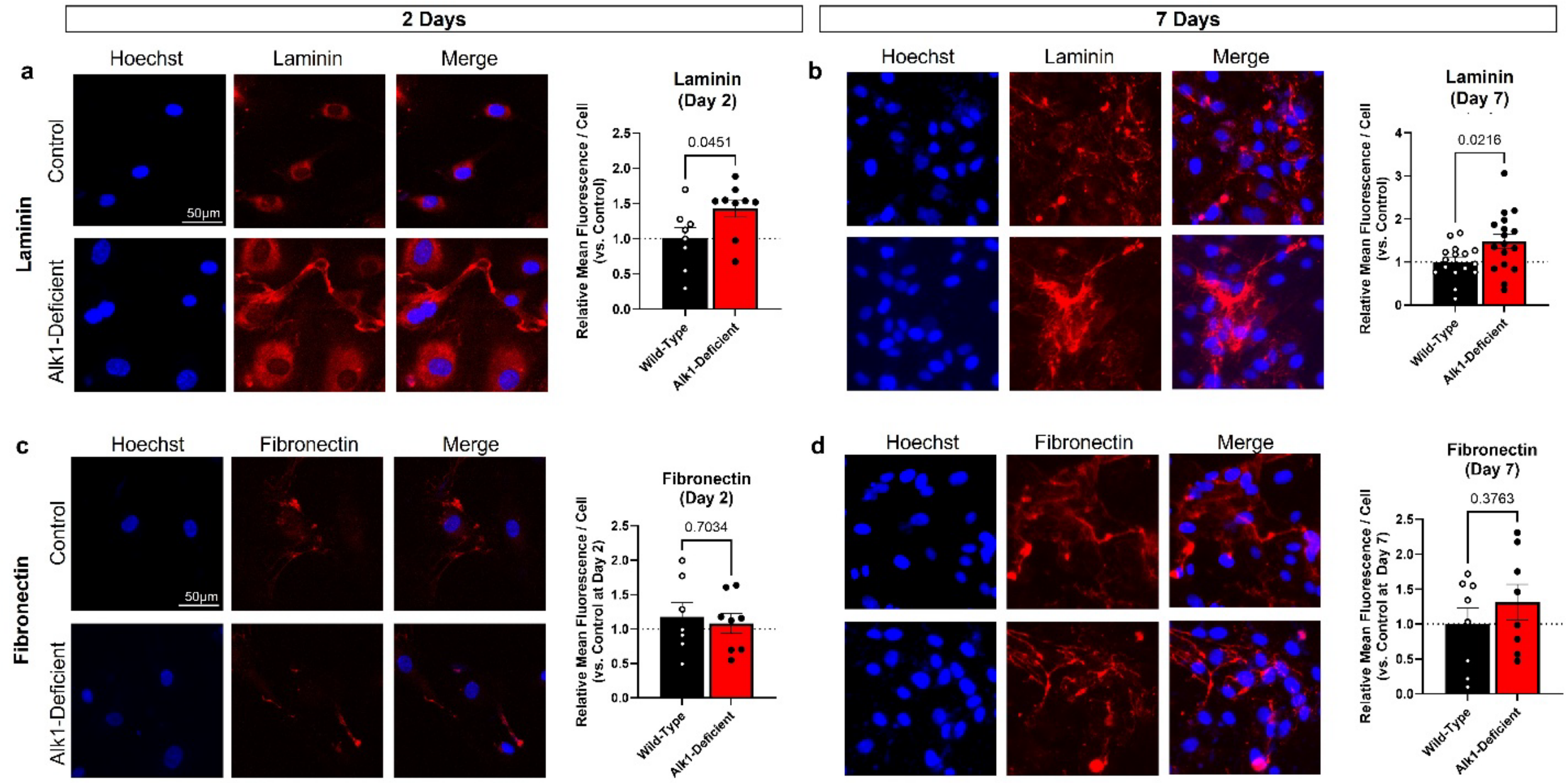

Supplementary Figure 13. Alk1-deficiency increases expression of laminin, but not fibronectin. Pan-laminin expression is increased following **a)** 2 days and **b)** 7 days of Alk1-deficiency (via IPTG treatment of sh-*ACVRL1* EC) vs. (no IPTG) controls). (Sample size: Day 2: Wild-Type: n=8, Alk1-Deficient: n=9, Day 7: Wild-Type: n=16, Alk1-Deficient: n=17 images. Statistics: Unpaired Students' t-Test). No significant differences in fibronectin expression were observed at **c)** 2 days or **d)** 7 days. (Sample size: Day 2: Wild-Type: n=7, Alk1-Deficient, n=8, Day 7: n=8 images per group, Statistics: Unpaired Students' t-Test). All plotted data are presented as mean values ± standard error.

	Score	Description
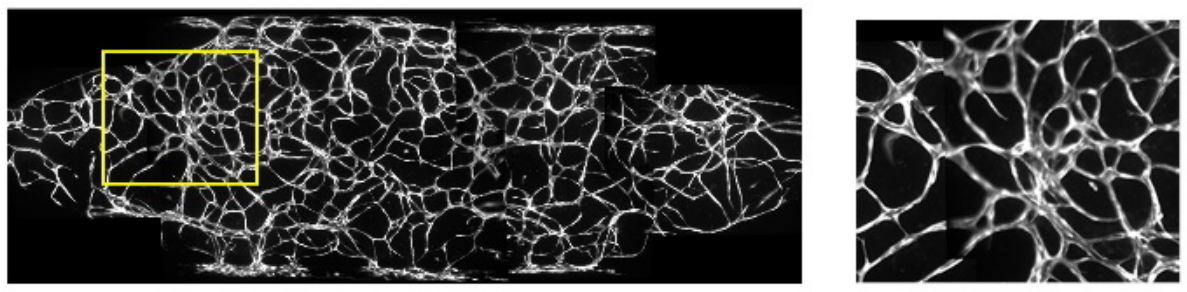	0	No lesions evident (i.e. vessel diameter appears mostly uniform throughout).
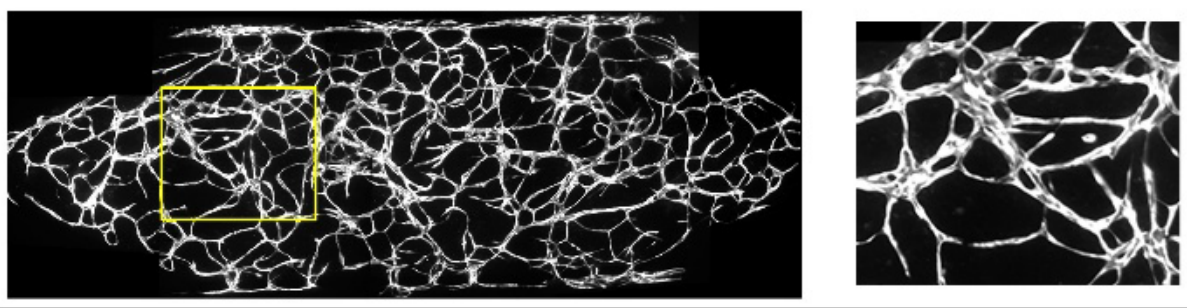	1	Dilated vessel lesions, where vessel morphology still appears mostly linear or tubular.
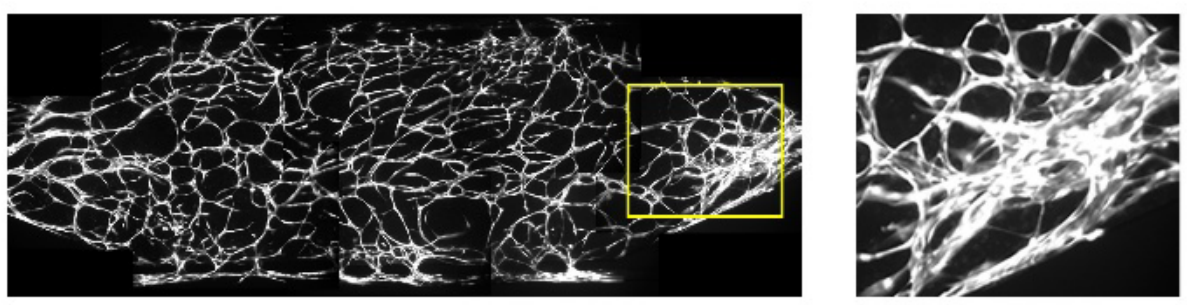	2	Dilated vessel lesions, where vessel morphology appears like a tangle or cluster.
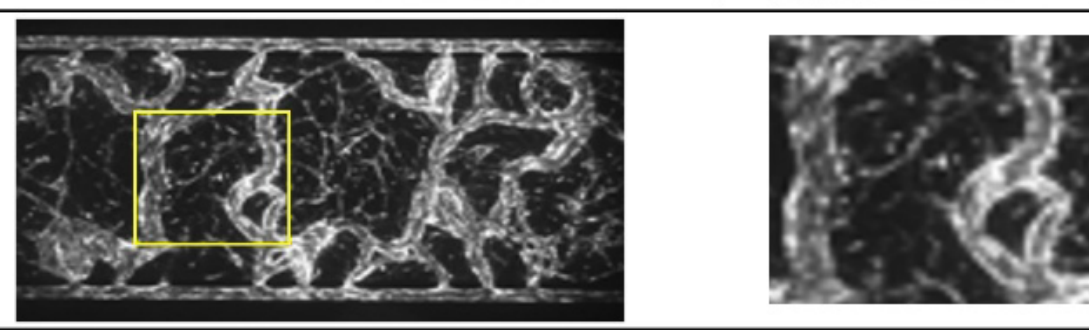	3	Dilated shunts that appear enlarged along majority of vessel length, and that connect top and bottom media channels evident.

Supplementary Figure 14: Vessel Disorganization Score. Scoring table and sample training images for vessel disorganization score analysis.
