# Supplementary material for: A microphysiological system HHT-on-a-chip platform recapitulates patient vascular lesions
Source: Nat Commun. 2026 Aug 13;17:9719. doi: 10.1038/s41467-026-76448-w (PMC13562586; doi:10.1038/s41467-026-76448-w)
Supplement: Supplementary file 2 [file 41467_2026_76448_MOESM2_ESM.pdf]

Description of additional Supplementary files

Supplementary data 1: Differentially-expressed genes (DEGs) in Diseased EC vs. all other EC
