# Supplementary material for: A microphysiological system HHT-on-a-chip platform recapitulates patient vascular lesions
Source: Nat Commun. 2026 Aug 13;17:9719. doi: 10.1038/s41467-026-76448-w (PMC13562586; doi:10.1038/s41467-026-76448-w)
Supplement: Supplementary file 4 [file 41467_2026_76448_MOESM5_ESM.pdf]

nature portfolio

Peer Review File

A Microphysiological HHT-on-a-Chip Platform Recapitulates Patient Vascular Lesions

Corresponding Author: Dr Christopher Hughes

This file contains all reviewer reports in order by version, followed by all author rebuttals in order by version.

Version 0:

Reviewer comments:

Reviewer #1

(Remarks to the Author)
Comments:
In this paper, the authors have developed a microfluidic HHT model validated with key feature such as hyperdense vasculature and AVM. It is noteworthy that AVM was successfully recreated through the co-culture of wild-type and transduced endothelial cells. Overall, the manuscript is well organized and clearly written. Here are some major and minor comments to the authors.

Major:
1. Authors have mentioned some hypothesis regarding lesion formation dominated by single clone of HHT-mutant EC expansion. Was there any difference in proliferation rate of wild-type or transduced HUVECs?
2. Authors have shown 70kDa dextran perfusion through patent lumen. Is there any difference in permeability values?
3. Is there a possibility to establish a link between the effects of pazopanib and the results of scRNA-seq?
4. How will the AVM develop when shear stress level increase globally? (Maybe by increasing the overall hydrostatic pressure level of both inlet).
5. Supplementary Figure 1 data seems to have only 6 days of data in E-I due to the decrease in siRNA effect, but if the shRNA effect was maintained for 14 days in the Figure 1 experiment, shouldn't the duration of the D-H experiments also be increased? Furthermore, the results of the 6-day experiments are weaker than the siRNA experiments, and the significance changes significantly from day to day, so perhaps shouldn't the experiments be monitored for more than 6 days?
6. ACVRL1 expression is much reduced in the shRNA experiment compared to the siRNA experiment. How could the authors explain that the significance is lower than siRNA case?
7. Some results are only up to day 6 and others are analyzed until day 14. Can the authors explain the reason for this difference"
8. Are there any differences in terms of basement membrane secretion or other protein, cytokine production due to the disease conditions or differences in cells?
9. The authors assumed steady-state model to calculate the flow and shear stress inside the vessel. However, hydrostatic pressure will gradually along time. Is it still possible to assume constant pressure head as an initial condition?

Minor:
1. Erratum in (Figure 6.G left column): Alk1-Intact EC >> Alk1-Deficient EC
2. In Figure 5, it would be good to add the significance between Alk1-, DMSO vs. Alk1-, Pazopanib to strengthen the validation of the effect of Pazopanib.
3. COMSOL simulation variables should be listed.

Reviewer #2

(Remarks to the Author)
In the study by Fang et al, a novel organ-on-a-chip model of Hereditary Hemorrhagic Telangiectasia (HHT), a rare congenital vascular disorder, was developed using the Vascularized Micro-organ (VMO) platform. This model, termed HHT-VMO, accurately mimics the vascular malformations (VMs) observed in HHT patients, including telangiectasias and arteriovenous malformations (AVMs). The researchers employed inducible ACVRL1-knockdown in human endothelial cells to control Alk1 expression, leading to VM development over several days. Notably, experiments revealed that AVM-like

lesions could consist of both Alk1-deficient and Alk1-intact cells, indicating potential cell non-autonomous effects. Single-cell RNA sequencing suggested that microvessel pruning and loss of PDGFB contribute to AVM formation, and the VEGFR inhibitor pazopanib was effective in blocking lesion formation, consistent with its positive effects in HHT patients. This model offers significant insights into HHT disease mechanisms and potential therapeutic approaches. The work is novel and thoroughly conducted. I only have few small suggestions for improvement.

1. I would suggest adding an overall schematics that shows the overview of the experimental system, the timelines and the approach. This will hopefully help to readers follow the manuscript easier.
2. Please explain better how the dose of pazopanib was selected.
3. Since human lung fibroblasts were used in co-culture, please comment if identified smooth muscle cells are actually myofibroblasts.
4. PDGFB signaling was identified from snRNAseq as one of the key disrupted pathways contributing to the phenotype development. Could the phenotype be fully or partially rescued by promotion of PDGFB signaling via growth factor supplementation or other approaches?

Reviewer #3

(Remarks to the Author)
In this manuscript, Hughes and colleagues describe a microphysiological platform to study a rare hereditary vascular disease, HHT-VMO. To effectively recapitulate the loss of ACVRL1 observed in HHT patients, the authors established an shRNA-mediated knockdown model in HUVECs. The authors demonstrate that both short-term and long-term knockdown of ACVRL1 results in hyper-dense microvascular network formation. This hyper-dense microvascular network can be reversed with the VEGFR2 inhibitor, pazopanib. The authors further show that the formation of enlarged vessels, reminiscent of vessel malformations, required (1) redesigning the microfluidic platform to support higher wall shear stress and (2) enriching for ACVRL1-deficient ECs. Strikingly, wall shear stress of 5 dynes/cm² supported the formation of vascular shunts in ACVRL1-deficient ECs, comprising both intact and deficient ECs, similar to what is found in patients. The authors next performed single-cell RNA sequencing of HHT-VMO constructs and identified a cluster of ECs (EC2) that was enriched in ACVRL1-deficient VMOs. Analysis of differentially expressed genes in the EC2 cluster showed enrichment in genes associated with vascular remodeling.

This is an important study that shows for the first time the utility of a microphysiological system to model vascular malformations, which could be applied not only to HHT but also other vascular diseases, of interest to a broad scientific community. The authors use an elegant approach with IPTG-inducible shRNA-mediated knockdown of ACVRL1 that allows tight control over the timing of the knockdown. They show that shRNA-mediated knockdown results in a more robust loss of ACVRL1 compared to siRNA-mediated knockdown, with a similar hyper-dense microvascular network formation. Additionally, the authors demonstrate that the phenotype can be rescued with pazopanib.

The manuscript is well-written, and most conclusions are well-supported experimentally. However, the manuscript would benefit from addressing the comments mentioned below.

Major comments:
1. ACVRL1 knockdown with IPTG-sensitive shRNA is shown in endothelial cells in monolayer; it would be beneficial to determine targeting efficiency in the VMO platform.
2. Does high and low flow conditions affect the expression of other flow-sensitive genes in ECs in addition to EphrinB2, such as KLF4?
3. How was the concentration of pazopanib determined? It would be helpful to demonstrate that 100 nM pazopanib effectively impacts VEGFR signaling.
4. Do ALK1-deficient ECs exhibit increased proliferation? An EdU assay could provide insights.
5. Computational modeling is used to determine shear stress under low and high flow conditions. However, it would be useful to perform particle image velocimetry to validate these findings and determine experimental shear stress in microvascular segments.
6. Please correct: there is an increase in vessels >100 µm and not >500 µm. It would be interesting if the authors could specify the the dimensions of AVMs?
7. Can pazopanib also prevent shunt formation?
8. Is there higher proliferation of ECs in the shunts, or an increase in cell size?
9. In Figure 7, it would be beneficial to characterize EC clusters 0, 1, and 3 in addition to cluster 2.
10. Figure 7E shows that many endothelial genes are downregulated. Are there any deregulated KEGG pathways? The presentation is unusual, as there are only 2-4 genes for each pathway.
11. It would be helpful to quantify the number of ECs in the EC dataset. Are there differences between ACVRL1 intact and deficient HHT-VMOs?
12. The authors mentioned that protein levels were reduced by immunofluorescence. The methods section also describes western blot, but no data for protein expression are shown by western blot. Please clarify, especially since ACVRL1 is hard to detect by WB.
Addressing these comments would strengthen the manuscript and provide a more comprehensive understanding of the findings.

Reviewer #4

(Remarks to the Author)
The main important significance of the manuscript NCOMMS-24-36005, is the attempt to establish a vascular organoid with a vascular organ on a chip device. This is the first important point, to create an "organoid" which may be useful as vascular organ (VMO), and maybe become a replacement for animal models which is a desirable objective for future research. The second point, that the device may be able to reproduce the pathological singularities of a vascular rare disease, hereditary hemorrhagic telangiectasia (HHT). In the third place, to use the VMO and the HHT modelisation, as a platform for screening therapeutic candidates for the disease.
The significance in the field of HHT is very high, since the development of lesions within an in vitro system, implies to throw light on the mechanisms leading to the vascular malformations observed in HHT, either, minor (increase of vascular density, abnormal vasculature), or major (arteriovenous direct connections in organs). On the other hand, this device maybe useful for other vascular diseases, not restricted to HHT. It could mimick, under the due conditions, atherosclerosis, or thrombotic events. Moreover, due to the fact that it is not easy to reproduce the HHT lesions in animal models, the possibilites of using an in vitro organoid for vasculature, seems exciting.

The work seems to support the conclusions and claims, but need additional evidences, which probably the authors may provide by revising the manuscript to improve the work.
First of all, a change on the results would be recommended. A section devoted to the device description, including figures like the Supplementary figure 8, with detailed explanation is needed. Also, in this same section, the use of the combination of HUVECs cells and fibroblasts to build the vessel structure needs an explanation. Why, the use of human lung fibroblasts? Are fibroblasts functionally able to replace pericytes, or smooth vasculature muscle cells (VSMCs) ? Why, VSMCs are not used?
Additional evidence is needed in relation to show more different figures of vascular malformations (shunts), and also, figures should be bigger and of higher quality. Most of the main figures contain many subsections, but all in all too small. Fig 1 needs to show enlarged size and more quality, in part B, almost imperceptible. The histograms are OK, but figures with cells should be enlarged with quality. Figure 2 should be enlarged in sections C, and D. Figure 3 show enlarged sections A, F and G. Figure 3 show enlarged sections A and B. In Figure 5, the treatment with Pazopanib, is showing some black lines which run from top to botton, almost in the middle of the picture (In panel A and panel B) Why? Enlarge the figure to see better A and B.
Fig 6, in the section A, at least 3 different cases of shunts should be presented. Panels C and D are dispensable, they may be commented in the text. In this way, panels A, B, may have room for more cases of shunts.
A main criticism is the fact of doing scRNA sequencing. Why is it necessary? This reviewer would suggest to make RNAseq of whole control and shACVRL1. Probably results may become more consistent, and with higher statistical significance. Authors should discuss the meaning of upregulation in matrix associated genes found in the sh ACVRL1 RNAseq, and the downregulation of several pro-angiogenic genes. Is this a transition process?

One mistake that should be corrected is in the abstract, line 60. It is not loss of these genes, but rather “a loss of function of the proteins encoded by the genes”
The methodology is sound and perfectly described. The only discordance is the fact of performing single cell RNA sequencing, instead of total control and silenced sh ACVRL1 RNAseq
The work meets expectancies in the HHT field as a step forward towards vascular organoids used as models, but more shunts and anomalies must be showed. The correction of the microvascular lesions after pazopanib treatment, should be shown in a more clear way, the images show in figure 5 are not enough. Another suggestion is that authors should also treat with other drug approved as orphan drug for HHT, and see also consistent improvement results as they try to show with pazopanib.
The description of the methodology seems to be enough to reproduce the results.

Other published models similar to this

- Engineered 3D vessel-on-chip using hiPSC-derived endothelial- and vascular smooth muscle cells (Stem Cell Reports) 2021 Sep 14;16(9):2159-2168. doi: 10.1016/j.stemcr.2021.08.003. Here, authors developed a multi-cell type 3D vessel-on-chip (VoC) model based entirely on human induced pluripotent stem cells (hiPSCs) in a fibrin hydrogel microenvironment, the hiPSC-derived vascular cells self-organized to form stable microvascular networks reproducibly, in which the vessels were lumenized and functional, responding as expected to vasoactive stimulation. HOWEVER, THEY DON'T REACH THE SECOND AND THIRD POINTS (reproduce pathological features of HHT and treat them with drugs).

This same group went further, in a second paper in the same journal Stem Cell Reports, when they were able to derive mutant ECs from iPSCs, and show a pathological defect in the vessels on a chip, a leakage: "Vascular defects associated with hereditary hemorrhagic telangiectasia revealed in patient-derived isogenic iPSCs in 3D vessels on chip" (Stem Cell Reports 2022 Jul 12;17(7):1536-1545. doi: 10.1016/j.stemcr.2022.05.022.
This dutch group which has developed vessels on a chip, is the closest idea to the one of the present manuscript. Differences: they work always with ECs derived from iPSCs, while in the present manuscript authors downregulate the expression of genes using HUVECs and using lentiviral particles to carry an inducible silencing shRNA.

The present manuscript is showning the development of a vascular model on a chip, the development of HHT like vascular malformations and the treatment of them with pazopanib (a drug used in clinical trials and HHT patients currently).

Version 1:

Reviewer comments:

Reviewer #1

(Remarks to the Author)
The authors have well addressed my concerns and I have no further comments.

Reviewer #2

(Remarks to the Author)
The authors have answered all of my questions. I have no further questions.

Reviewer #3

(Remarks to the Author)
I am satisfied with the authors' response to my and the other reviewers' comments, and I think the manuscript has been substantially improved. I also appreciate that the authors performed additional experiments with bevacizumab, although these experiments were not specifically requested by the reviewers.

I do have some reservations about how the new bevacizumab experiments are interpreted. The conclusion that established lesions are VEGF-independent is interesting, but the current experimental design does not fully separate the effect of lesion stage from the effect of drug choice. In other experiments on lesion formation, pazopanib was used to assess effects on lesion development, whereas bevacizumab was used to assess effects on established lesions. As a result, the lack of response in established lesions could reflect stage-specific VEGF independence, but it could also reflect differences between the two inhibitors, such as their mechanisms of action, antibody diffusion through the hydrogel in the microfluidic chips given the relatively large molecular weight of bevacizumab, or the duration of treatment. For this reason, testing pazopanib on established lesions would provide a more direct comparison with the other experiments on lesion formation and would more clearly address whether established lesions lose sensitivity to VEGF pathway inhibition.

I also think the mechanistic explanation based on reduced VEGFR1/2 expression in "Diseased EC" would benefit from further clarification. Given the authors' finding that lesion formation involves cell non-autonomy and recruitment of Alk1-intact endothelial cells, it is important to know whether these recruited cells also acquire a VEGFR-low state within established lesions. Clarifying whether "Diseased EC" refers to genotype, transcriptional state, or both would strengthen the proposed explanation and help determine whether it applies to the whole lesion or primarily to the mutant endothelial cell compartment.

Reviewer #4

(Remarks to the Author)
The HHT-VMO (Hereditary Haemorrhagic Telangiectasia – Vascularised Micro-Organ) model is an organ-on-a-chip platform developed to study the characteristic vascular lesions of hereditary haemorrhagic telangiectasia (HHT). This model uses human cells and microengineering to faithfully reproduce the vascular malformations observed in patients with HHT, enabling detailed analysis of the biology of the disease and the evaluation of potential drug treatments.

Significance to the Field and Related Fields:

The HHT-VMO (Hereditary Hemorrhagic Telangiectasia – Vascularized Micro-Organ) model represents a major advance in the study of vascular malformations, particularly for HHT. Its significance is supported by several key points:
First fully human, cell-based microphysiological model: Unlike most prior models that rely on genetically modified mice or zebrafish, the HHT-VMO uses primary human endothelial cells and microengineering to recapitulate patient-like vascular lesions (telangiectasias and AVMs) in vitro.Recapitulates patient pathology: The model reliably produces vascular malformations that closely resemble those seen in HHT patients, including both small and large lesions. This fidelity is not matched by previous in vitro models.Drug response mirrors clinical outcomes: The model demonstrates that pazopanib (a VEGFR inhibitor) prevents lesion formation, while bevacizumab (a VEGF inhibitor) does not resolve established lesions—findings that align with clinical observations in HHT patients.Mechanistic insights: The HHT-VMO allows precise control over gene knockdown and flow conditions, revealing that only a small proportion of Alk1-deficient cells can trigger lesions, and that there is a critical window during vessel formation when Alk1 is required. These insights are difficult to obtain in animal models.Scalability and real-time observation: The platform is adaptable to high-throughput formats, enabling drug screening and dynamic observation of lesion development.
Broader Impact
Translational research: The model bridges the gap between animal studies and human disease, supporting drug discovery and personalized medicine.Related fields: The approach can be adapted to study other vascular diseases, tumor angiogenesis, and organ-on-chip applications.
Comparison to Established Literature
Animal models: Previous research has relied heavily on mouse and zebrafish models (see references 10, 11, 15, 19, 20, 21, 23, 39). While these have provided valuable insights, they are limited by species differences and sporadic lesion formation.Other in vitro models: Orlova et al. used iPSC-derived EC from HHT1 patients in a vessel-on-a-chip, but did not observe the full spectrum of HHT-like lesions, likely due to residual gene expression and lack of a second-hit mutation (see Discussion).Recent AVM-on-a-chip models: Soon et al. (reference 57) modeled brain AVMs using mutant KRAS EC, but did not focus on HHT-specific mechanisms or recapitulate the full range of HHT pathology.Novelty: The HHT-VMO is the first to

combine human cells, precise genetic manipulation, and physiological flow to robustly model both telangiectasias and AVMs, and to demonstrate mosaicism and cell non-autonomous effects as seen in patient lesions.
Originality: The work is original in its design, methodology, and the depth of mechanistic and translational insights provided. It is the first to:
-Reproduce HHT lesions in a fully human, flow-adapted organ-on-chip system.Demonstrate the critical timing of Alk1 loss and the sufficiency of a minority of mutant cells for lesion formation.Show drug responses that mirror clinical outcomes in HHT patients.Relevant references for comparison:
Snellings et al., Am J Hum Genet 2019 (reference 13): Mosaicism in HHT lesions. Orlova et al., vessel-on-a-chip with iPSC-EC (discussed in the manuscript).Soon et al., Biomaterials 2022 (reference 57): AVM-on-a-chip.Larrivee et al., Dev Cell 2012 (reference 39): Flow and AVM formation in mice.Han et al., Angiogenesis 2014 (reference 20): VEGF neutralization in animal models.
Conclusion
The HHT-VMO work is highly significant and original, providing a new, human-relevant platform for studying HHT and vascular malformations. It advances the field beyond animal and previous in vitro models, offers mechanistic and translational insights, and is well-supported by and compared to the established literature.

To conclude the manuscript on my view is able to be published, as authors have answered the points raised by the reviewers, recognising limitations, like, the type of flow, the type of mutations, and the lack of interaction with other players in the physiology of a complete human being.
It is a first step as a proof of concept.

Open Access This Peer Review File is licensed under a Creative Commons Attribution 4.0 International License, which permits use, sharing, adaptation, distribution and reproduction in any medium or format, as long as you give appropriate credit to the original author(s) and the source, provide a link to the Creative Commons license, and indicate if changes were made.
In cases where reviewers are anonymous, credit should be given to 'Anonymous Referee' and the source.
The images or other third party material in this Peer Review File are included in the article's Creative Commons license, unless indicated otherwise in a credit line to the material. If material is not included in the article's Creative Commons license and your intended use is not permitted by statutory regulation or exceeds the permitted use, you will need to obtain permission directly from the copyright holder.
To view a copy of this license, visit https://creativecommons.org/licenses/by/4.0/
